# Interchangeability of class I and II fumarases in an obligate methanotroph *Methylotuvimicrobium alcaliphilum* 20Z

**DOI:** 10.1371/journal.pone.0289976

**Published:** 2023-10-26

**Authors:** Oleg I. Melnikov, Ildar I. Mustakhimov, Alexander S. Reshetnikov, Maxim V. Molchanov, Andrey V. Machulin, Valentina N. Khmelenina, Olga N. Rozova

**Affiliations:** 1 Federal Research Center “Pushchino Scientific Center for Biological Research of the Russian Academy of Sciences”, G.K. Skryabin Institute of Biochemistry and Physiology of Microorganisms, Russian Academy of Sciences, Pushchino, Moscow Region, Russia; 2 Institute of Theoretical and Experimental Biophysics, Russian Academy of Sciences, Pushchino, Moscow Region, Russia; Federal University Dutse, NIGERIA

## Abstract

The methanotrophic bacterium *Methylotuvimicrobium alcaliphilum* 20Z is an industrially promising candidate for bioconversion of methane into value-added chemicals. Here, we have study the metabolic consequences of the breaking in the tricarboxylic acid (TCA) cycle by fumarase knockout. Two fumarases belonging to non-homologous class I and II fumarases were obtained from the bacterium by heterologous expression in *Escherichia coli*. Class I fumarase (FumI) is a homodimeric enzyme catalyzing the reversible hydration of fumarate and mesaconate with activities of ~94 and ~81 U mg^-1^ protein, respectively. The enzyme exhibited high activity under aerobic conditions, which is a non-typical property for class I fumarases characterized to date. The calculation of *k*_cat_/*S*_0.5_ showed that the enzyme works effectively with either fumarate or mesaconate, but it is almost four times less specific to malate. Class II fumarase (FumC) has a tetrameric structure and equal activities of both fumarate hydration and malate dehydration (~45 U mg^-1^ protein). Using mutational analysis, it was shown that both forms of the enzyme are functionally interchangeable. The triple mutant strain 20Z-3E (ΔfumIΔfumCΔmae) deficient in the genes encoding the both fumarases and the malic enzyme accumulated 2.6 and 1.1 mmol g^-1^ DCW fumarate in the medium when growing on methane and methanol, respectively. Our data suggest the redundancy of the metabolic node in the TCA cycle making methanotroph attractive targets for modification, including generation of strains producing the valuable metabolites.

## Introduction

Fumarase, or fumarate hydratase (EC 4.2.1.2), is an enzyme of the canonical tricarboxylic acid cycle (TCA). It catalyzes the reversible hydration of fumarate to (S)-malate. There are two non-homologous classes of the enzyme. Class I fumarases are represented by homodimeric or heterodimeric enzymes with molecular masses of ~120 kDa and 65 kDa, respectively [1, 2]. These fumarases contain an oxygen-sensitive catalytic [4Fe-4S] cluster, which is readily oxidized to an inactive [3Fe-4S] cluster [3, 4]. Unlike the Fe-S clusters in other proteins, O^2-^ probably has a direct access to the Fe-S clusters of the hydrolyase. In addition, class I fumarases are characterized by a wide substrate specificity and able to catalyze S,S-tartrate/oxaloacetate and mesaconate/S-citramalate interconversions with varying catalytic efficiencies [2, 4]. The fumarases of class I are found in all three domains of life but are absent in *Homo sapiens* or *Saccharomyces cerevisiae* [2]. The thermophilic bacteria and hyperthermophilic archaea possess thermostable heterodimeric fumarases [1, 5]. In *Escherichia coli*, there are thermolabile homodimeric fumarases A and B (FumA and FumB) from class I, sharing a high degree of sequence similarity and having similar catalytic properties, but they are expressed under different physiological conditions [6]. The third form of class I fumarase, FumD, has been found in many pathogenic bacteria, including the *E*. *coli* strain O157:H7 [2].

Class II fumarases consist of four identical 50 kDa subunits. These enzymes are oxygen tolerant and strongly specific to fumarate/malate. Class II fumarases can be found in many pro- and eukaryotic organisms, including *E*. *coli*, and are referred to as FumC. Unlike Fum A and B, *E coli* Fum C retained its activity after heating the cell extract at 50 for 80 min [7]. The signature sequence (GSSxMPxKxNPxxxE) lying between Gly 317 and Glu 331 (relative to *E*. *coli* FumC) is found in class II fumarases, as well as in aspartase, argininosuccinate lyase, adenylosuccinate lyase, cis-muconate lactonizing enzyme, and δ–crystallin in one broad superfamily [8]. Many bacteria have fumarases of both classes.

**Fig 1:**
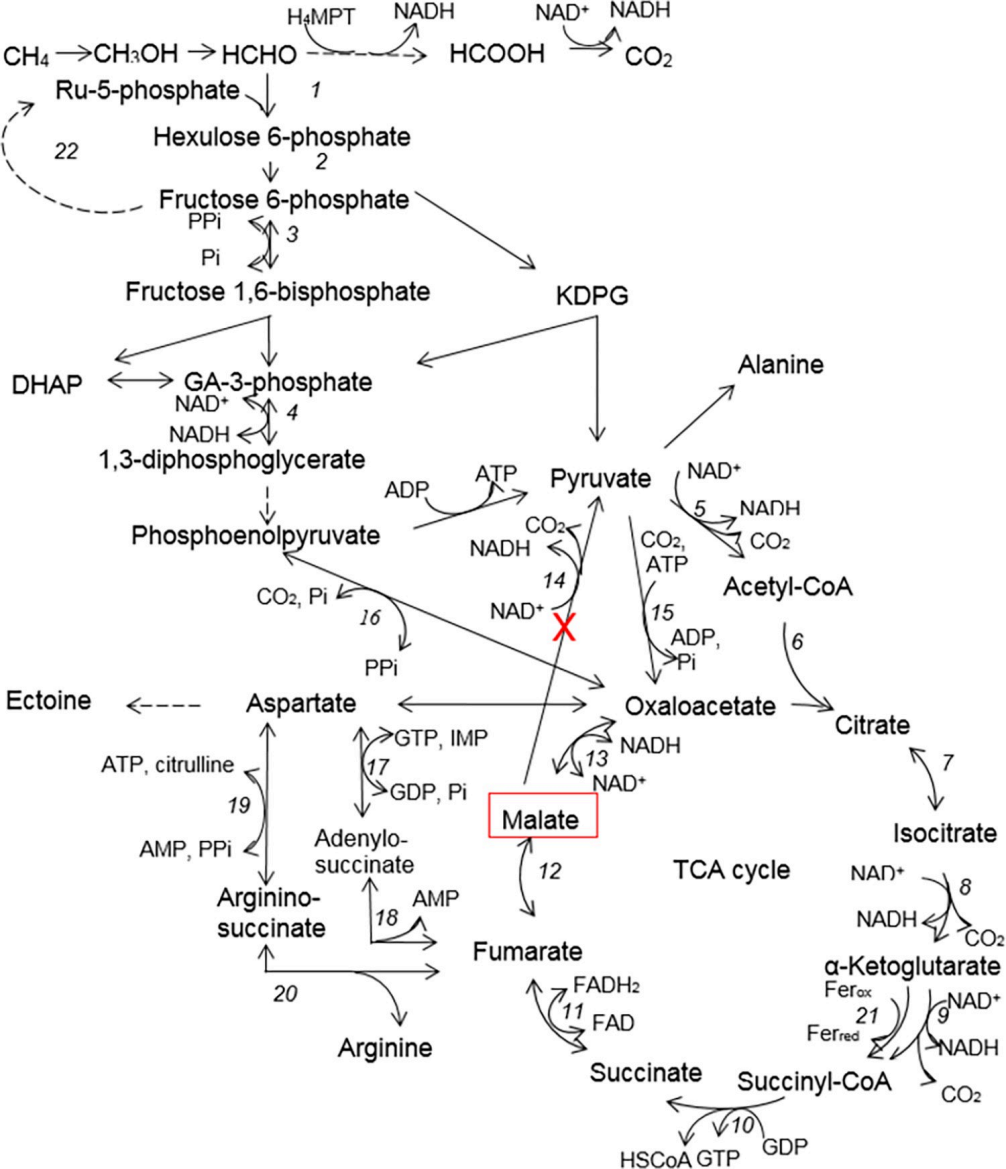
The central metabolism of *M*. *alcaliphilum* 20Z. 1 –hexulose 6-phosphate synthase, 2 –phosphohexuloisomerase 3 –PPi-dependent 6-phosphofructokinase, 4 –glyceraldehyde-3-phosphate dehydrogenase, 5 –pyruvate dehydrogenase complex, 6 –citrate synthase, 7 –aconitase, 8 –isocitrate dehydrogenase, 9 – α-ketoglutarate dehydrogenase complex, 10 –succinyl CoA synthetase, 11 –succinate dehydrogenase, 12 –fumarase, 13 –malate dehydrogenase, 14 –malic enzyme, 15 –pyruvate carboxylase, 16 –PPi-dependent phosphoenolpyruvate carboxykinase, 17 –adenylosuccinate synthase, 18 –adenylosuccinate lyase, 19 –argininosuccinate synthase, 20 –argininosuccinate lyase, 21 – α-ketoglutarate: ferredoxin oxidoreductase, 23 –pentose phosphate pathway.

Methanotrophs are a group of bacteria utilizing methane as a sole carbon and energy source. The representatives of gammaproteobacterial methanotrophs (type I methanotrophs) are industrially promising bacteria. They use the ribulose monophosphate (RuMP) cycle as the main pathway of methane assimilation (Fig 1). Methanotrophs generate energy from methane oxidation to CO_2._ Previously, it was believed that the TCA cycle in type I methanotrophs is incomplete due to the absence of α-ketoglutarate dehydrogenase activity, as a result of which it plays an predominant anabolic function [9]. However, in the recent studies, it was shown that in halotolerant methanotroph *Methylotuvimicrobium buryatense* 5GB1, the TCA cycle is complete when grown on methane and it is incomplete in methanol grown culture [10, 11]. Therefore, features of the TCA cycle functioning in aerobic methanotrophs require clarification. Earlier, in *M*. *alcaliphilum* 20Z, we have disrupted the *mae* gene encoding the malic enzyme, that resulted in an increase in the malate level in methanol-grown cells [12]. Here we have characterized two fumarases (FumI and FumC) in *M*. *alcaliphilum* 20Z. The *fumI* and *fumC* knockout mutants were based on the *M*. *alcaliphilum* Δmae strain. We have found out that two fumarases are interchangeable in the methanotroph. The triple mutant strain with the *ΔfumIΔfumCΔmae* genotype accumulated in the medium fumarate 2.6 and 1.1 mmol g^-1^ DCW during the growth on methane and methanol, respectively. Phylogenetic analysis has shown the limited distribution of class I fumarase among methanotrophs.

## Materials and methods

### Bacteria and growth conditions

*M*. *alcaliphilum* 20Z (VKMB-2133, NCIMB14124) was grown at 30°C in a liquid nitrate mineral salt medium P containing 1% (w/v) NaCl and 0.1 M sodium carbonate buffer (pH 9.0) [13] in the methane–air (1:1) mixture or in the presence 0.3% methanol. Selective medium P contained 100 μg mL^-1^ kanamycin. *Escherichia coli* strain BL21 (DE3) (Novagen) was grown at 37°C in a selective Luria–Bertani (LB) medium containing 50 μg mL^-1^ kanamycin or ampicillin, agar or broth with constant shaking (150 rpm).

### Gene cloning and purification of recombinant enzymes

The *fumC* gene 1404 bp encoding class II fumarase (or FumC) in *M*. *alcaliphilum* (CCE21981.1) was amplified by PCR using primers FumC-F and FumC-R (S1 Table) designed from the sequence available in the GenBank (accession number NC_016112). Amplification of the *fumI* gene encoding the putative class I fumarase in *M*. *alcaliphilum* 20Z was carried out using primers FumI-F and FumI-R. For expression, each of the genes was ligated into a vector pET22b(+) and the resulting vectors pET22b:fumC and pET22b:fumI were transformed into *E*. *coli* BL21 (DE3). The enzyme expression was induced at OD_600_ of 0.6–0.8 with 0.5 mM IPTG. After 15 h of growth at 18°C, cells were harvested by centrifugation (30 min at 8°C and 5000 g) and stored at –20°C. The His_6_-tagged proteins were purified by metal chelate chromatography on a Ni^2+^-nitrilotriacetic acid (Ni-NTA) column as described earlier [14]. The purified FumC was stored in 40% (v:v) glycerol at –20°C, whereas class I fumarase was stored in a mixture containing 50 mM Tris-HCl (pH 8.0), 25 mM (NH_4_)_2_SO_4_, 25 mM FeSO_4_, 25 mM dithiothreitol (DTT), and 50% (v:v) glycerol at 4°C [15]. To obtain an oxygen-free FumI, purification was carried out with buffer solutions pre-boiled (approximately 30–60 sec) in a microwave oven. The purified enzyme was purged with nitrogen in a sealed vial.

### Determination of the molecular masses of FumI and FumC

The molecular masses of the recombinant enzymes were estimated using gel filtration chromatography on a XK 16/100 Superdex 200 column (GE Healthcare) equilibrated with 0.02M Tris-HCl (pH 7.0) containing 0.5 M NaCl. The flow rate was 1 mL min^-1^, and the proteins were monitored at 280 nm. The protein standards (Merck, Germany) were carbonic anhydrase (29 kDa), albumin (66 kDa), alcohol dehydrogenase (150 kDa), β-amylase (200 kDa), and apoferritin (443 kDa).

### Enzyme activities assays

The activity of FumI in the hydration reaction was assayed by recording malate formation from fumarate in 1 mL of the reaction mixture containing 0.05 M Tris-HCl, pH 8.5; 5 mM MgCl_2_; 50 mM KCl; 0.3 mM NADP^+^ and 30 μg of FumI. 20U of NADP-dependent malic enzyme from *Methylosinus trichosporium* was used as a coupling enzyme [16]. The activity of FumC was measured using 0.05 M Tris-HCl (pH 8.0) and 140 μg FumC. The reaction was started with 1 mM fumarate and NADP^+^ reduction was recorded at 340 nm. This method was used to test various metabolites as potential effectors for the enzymes and to determine fumarase activity in cell free extracts of the methanotroph. To determine the pH and temperature optima and kinetic characteristics, the activities of each fumarase were measured without the coupling enzyme. The reaction mixture for FumI (1 mL) contained: 0.05 M Tris-HCl, pH 8.5 and up to 30 μg of the enzyme. The reaction mixture for FumC contained 0.05 M Tris-HCl, pH 8.0, and up to 140 μg FumC. The reaction was started with 1 mM fumarate or mesaconate; the hydration reaction was registered spectrophotometrically at 250 nm (ε_fumarate_ = 2.4 mM^-1^cm^-1^, ε_mesaconate_ = 2.26 mM^-1^cm^-1^) [15].

The fumarase activity in the malate dehydration reaction was assayed at 250 nm in 50 mM Tris-HC1 (pH 8.5) containing 1 mM malate, ~50 μg FumI, or ~ 150 μg FumC.The effect of pH on fumarase activity was studied using the following buffers (50 mM): CHES-NaOH (pH 8.5–10.0), glycine-NaOH (9.0–10.5), Tris-HCl (pH 7.0–9.0), K-phosphate (6.0–8.0), MES-NaOH (5.0–7.0). The potential effectors tested were: 5 mM for glucose-6-phosphate, fructose-6-phosphate, fructose-1,6-bisphosphate; 1 mM for phosphoenolpyruvate, oxaloacetate, citrate, pyrophosphate (PPi), lactate, α-ketoglutarate, serine, hydroxypyruvate, glycine, pyruvate, succinate. Protein concentration was estimated by a modified Lowry method using bovine serum albumin as a standard.

### Mutant generation

Since in the previous study we obtained a kanamycin resistant strain *M*. *alcaliphilum* mae^*-*^, for this work we obtained a markerless strain *M*. *alcaliphilum Δmae* based on a counter selection system with the *sacB* gene encoding levansucrase [17]. Using two pairs of primers mae-up-F/mae-up-R and mae-dw-F/mae-dw-R (S1 Table), the upstream and downstream regions (~700 bp) of the *mae* gene were amplified with methanotrophic gDNA and cloned into the vector pCMSacB at restriction sites BglII/NdeI and ApaI/SacI, respectively. *E*. *coli* S17-1 transformed by the resulting vector pCMSacB:mae-up-down was used to conjugation *M*. *alcaliphilum* 20Z. The kanamycin resistant clones were selected on 2.5% (w/v) sucrose. In the clones, which lost kanamycin resistance, the full deletion of *mae* gene was checked by PCR (S1 Fig).

Similarly, the pair of primers FumC-F-up/FumC-R-up and FumC-F-down/FumC-R-down was used for the *fumC* gene deletion and FumI-F-up/FumI-R-up and FumI-F-down/FumI-R-down was used for the *fumI* gene deletion (S1 Table). To obtain a double/triple mutant, the vector pCMSacB:fumC-up-down or pCMSacB:fumI-up-down were introduced into *M*. *alcaliphilum* Δmae cells by conjugating with *E*. *coli* S17-1. Then the clones were selected on sucrose.

### Analysis of extracellular metabolites

*M*. *alcaliphilum* and the mutant strains were grown up to the stationary growth phase in 750-ml flasks containing 200 mL of a mineral salt medium supplemented with 1% NaCl under stirring. 450 μl of cell-free culture liquid was dried and then dissolved in 570 μL of H_2_O followed by the addition of 30 μL of 4 M 3-trimethylsilyl [2,2,3,3-2H4] propionate (TSP) solution in D2O. One-dimensional (1D) and two-dimensional (2D) nuclear magnetic resonance spectra were recorded with a Bruker 600 AVANCE III NMR spectrometer (Bruker BioSpin, Reinstetten, Germany) at an operating frequency of 600 MHz, at 298 K, with a spectral width of 24 ppm and a 90-degree pulse of 11 μs. Free induction decay (FID) was recorded for aq = 3.42 s at 96 k points. When taking NMR spectra, the time between scans was 10 s, which was sufficient for the relaxation of protons of metabolites observed. Two-dimensional spectra of the homonuclear (1H–1H) COSY spin–spin correlation (sequence COSYGPPRQF) for the samples were recorded over the entire signal regions. The time delay between COSY pulses was 1 s, the amount of data was about 2048/512 points. To achieve an acceptable signal-to-noise ratio for 1D experiments, the number of accumulations was 128 and from 4 to 32 for 2D spectra. Chemical shifts were calibrated using the TSP signal at 0.00 ppm, which served as an internal standard. The spectra were processed and the integrals were calculated using the TOPSPIN program (Bruker). To interpret the spectra and to assign the signals of metabolites, we used 1D and 2D NMR spectra, as well as the spectral database in the AMIX software (Bruker).

### Model and features of FumI

The structure of a homodimer of class I fumarase from *M*. *alcaliphilum* (UniProt ID G4T1T1) was predicted using AlphaFold2 in the homodimer mode (https://alphafold.ebi.ac.uk/ [18]. The position of the iron-sulfur cluster was determined according to the structure of fumarase 2 from *Leishmania major* (UniProt ID E9AE57; PDB ID 5L2R). The resulting tertiary structure was visualized using the PyMOL v.2.5 molecular graphics system (https://pymol.org/2/). Fumarase parameters were assessed using ProtParam (https://web.expasy.org/protparam/) and PONDR v. VLXT (http://www.pondr.com/).

### Sequence analysis

The sequences from NCBI, IMG/MER and MicroScope databases (http://www.ncbi.nlm.nih.gov, https://mage.genoscope.cns.fr/, https://img.jgi.doe.gov/) were obtained by BLAST searches. The alignments of amino acid sequences of different fumarases and the phylogenetic analysis were performed using MEGA 6 and the Neighbor-Joining model [19]. Minor corrections in the alignments were done manually.

## Results

### Purification of the *M*. *alcaliphilum* fumarases

**Fig 2:**
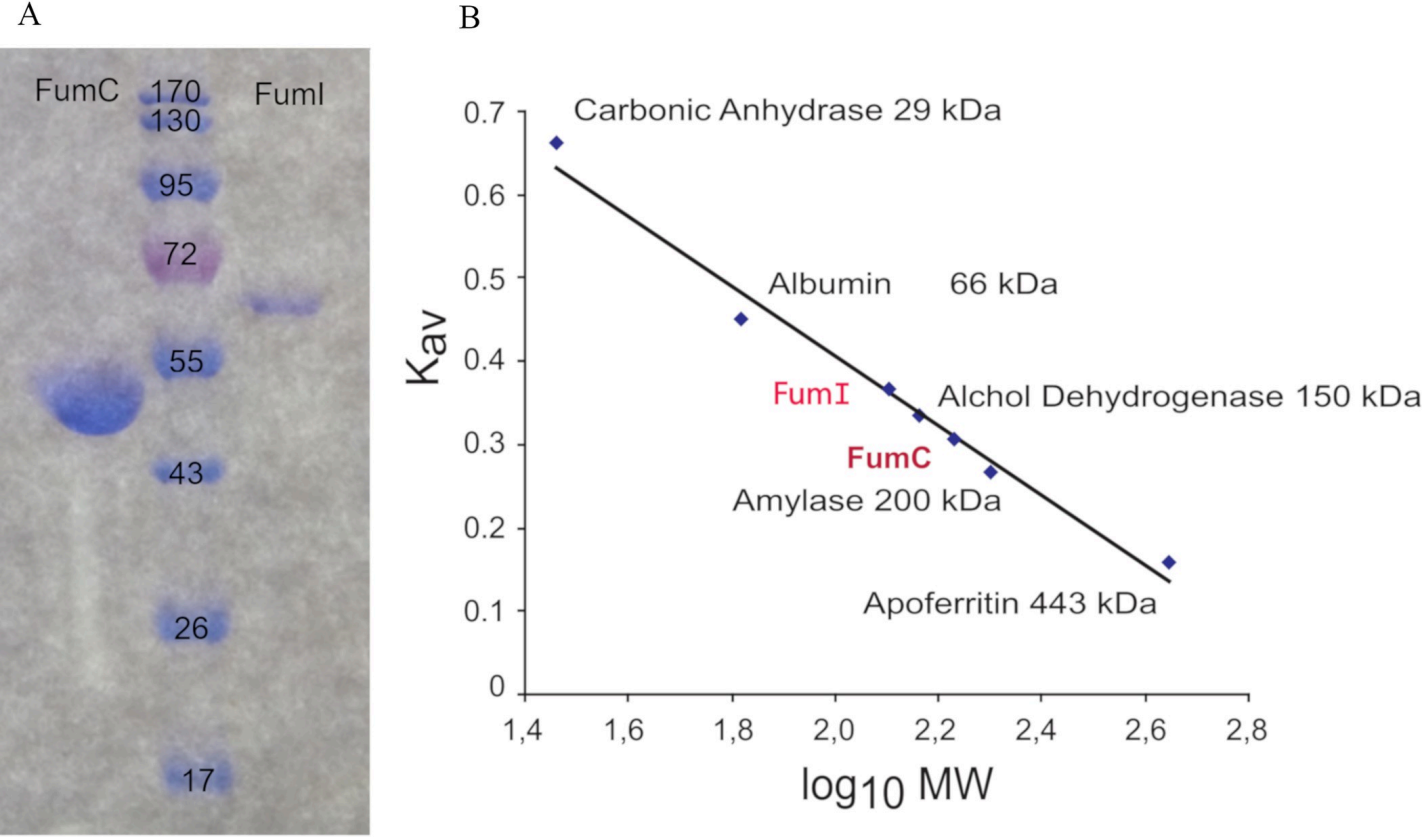
Purification and determination of oligomeric state of the recombinant FumI and FumC from *M*. *alcaliphilum* 20Z. (A) The purity of the proteins was determined by 12% SDS-PAGE. M, molecular mass of the markers. (B) Molecular mass determination by gel filtration chromatography.

The *fumI* and *fumC* genes from *M*. *alcaliphilum* 20Z were cloned in the pET22b(+) vector and expressed in the cells of *E*. *coli* BL21 (DE3). The His_6_-tagged proteins purified by Ni^2+^-bounded affinity chromatography were homogeneous according to electrophoresis under denaturing conditions. The molecular mass of a FumC subunit (~50 kDa, Fig 2A) was close to the value predicted from the coding sequence (50.1 kDa). The electrophoretic mobility of FumI corresponded to the molecular mass of a subunit ~60 kDa, which was slightly higher than the predicted value (55.2 kDa). According to gel filtration (Fig 2B), the native molecular mass of FumI was 130 kDa, indicating a dimeric structure of the protein. Such structure was found for other class I fumarases, which are homomeric. The molecular mass of FumC (184 kDa) corresponds to the tetrameric structure of the enzyme, similar to the previously characterized class II fumarases [20, 21].

### The kinetic properties and structural features of *M*. *alcaliphilum* FumI

**Fig 3:**
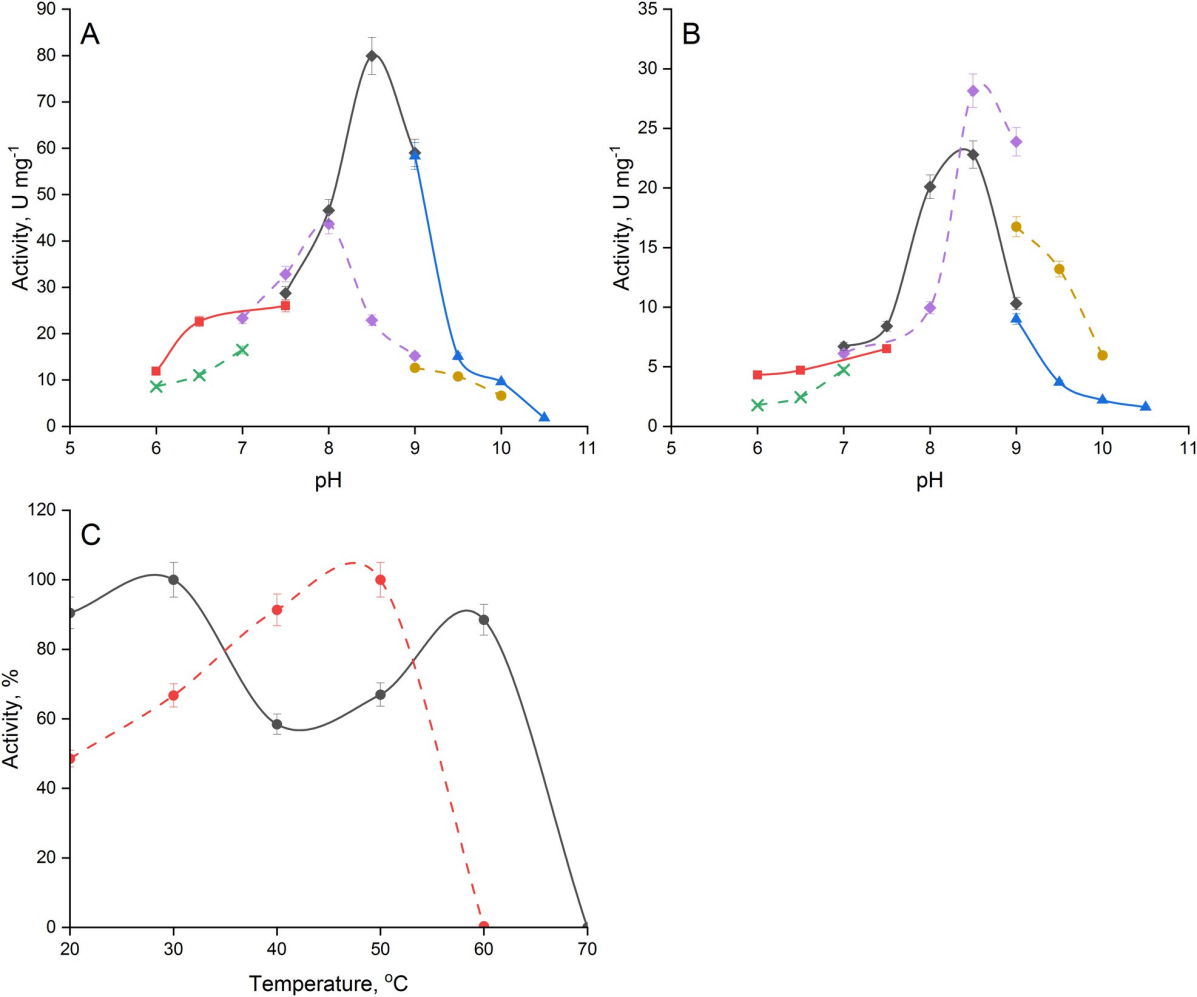
Effect of pH (A, B) and temperature (C) on the activity of FumI (solid line) and Fum C (dashed line) from *M*. *alcaliphilum* 20Z in the reactions of fumarate hydration (A, C) and malate dehydration (B): (diamond)–Tris-HCl, (square)–K-phosphate, (triangle)–Glycine-NaOH, (x)–MES-NaOH, (circle)–CHES-NaOH.

The aerobically purified enzyme was stabilized with an iron-sulfur mixture. Under aerobic conditions, the enzyme catalyzed fumarate hydration (with the activity of 94 ± 7 U mg^-1^ protein) and malate dehydration (50 ± 1 U mg^-1^ protein). FumI demonstrated the maximum activity at 30°C and pH 8.5 in either fumarate hydration or malate dehydration (Fig 3A, 3B). The fumarase activity at 60°C was almost as high as at 30°C (Fig 3C). The enzyme withstood a one-hour exposure at 30–50°C without the loss of activity but was completely inactivated after 30 min at 60°C. Previously, rapid inactivation of the FumA and FumB activities was shown during heating of *E*. *coli* cell extracts at 50°C [7]. The anaerobically purified FumI which was stabilized by the iron-sulfur mixture, catalysed fumarate hydration or malate dehydration 3.5 and 1.5 times slowly when aerobically purified enzyme.

The curves of substrate saturation in direct and reverse reactions did not obey the Michaelis–Menten kinetic equation (S2A, S2C, S2E Fig). Therefore, we used the *S*_0.5_ value with the Hill coefficient. Under optimal conditions at various substrate concentrations, the following *S*_0.5_ values were obtained: 0.28 ± 0.04 mM for fumarate (Hill’s coefficient *n* = 1.4 ± 0.2) and 0.55 ± 0.03 mM for malate (*n* = 1.7 ± 0.1). Similar to other class I fumarases, the enzyme from *M*. *alcaliphilum* hydrated mesaconate (2-methylfumarate) to 2-methylmalate ((S)-cytramalate). The activity with mesaconate was 81 ± 3 U mg^-1^, and *S*_0.5_ for mesaconate was 0.24 ± 0.02 mM (*n* = 1.4 ± 0.1). The calculation of *k*_cat_/*S*_0.5_ showed that fumarase was 4 times more specific to fumarate than to malate (Table 1). 1 mM succinate increased the FumI activity by about 1.5 times (S2 Table), and no other effectors were found.

**Table 1:** Kinetic parameters of *M*. *alcaliphilum* fumarases.

Enzyme Substrate	Class I fumarase					Class II fumarase (FumС)				
	*S*_0.5_, mM	V_max_, U mg^-1^	*k*_cat,_ s^-1^	*k*_cat_/*S*_0.5_, s^-1^ M^-1^	Specificity_: (_*k*_cat/_*K*_m_^fumarate^_) /(_*k*_cat/_*K*_m_^malate^_)_	*K*_m_(*S*_0.5_*), mM	V_max_, U mg^-1^	*k*_cat_, s^-1^	*k*_cat_/*K*_m_(*S*_0.5_)_,_ s^-1^ M^-1^	Specificity_: (_*k*_cat/_*K*_m_^fumarate^_) /(_*k*_cat/_*K*_m_^malate^_)_
Fumarate	0.28 ± 0.04	94 ± 12	0.2	7.1 x 10^2^	3.9	0.11 ± 0.004	45 ± 1	0.14	1.2 x 10^3^	1.2
Malate	0.55 ± 0.02	50 ± 1	0.1	1.8 x 10^2^		0.14 ± 0.01*	42 ± 3	0.13	1.0 x 10^3^	
Mesaconate	0.24 ± 0.2	81 ± 5	0.18	7.5 x 10^2^	4.2	-	-	-	-	-

**Fig 4:**
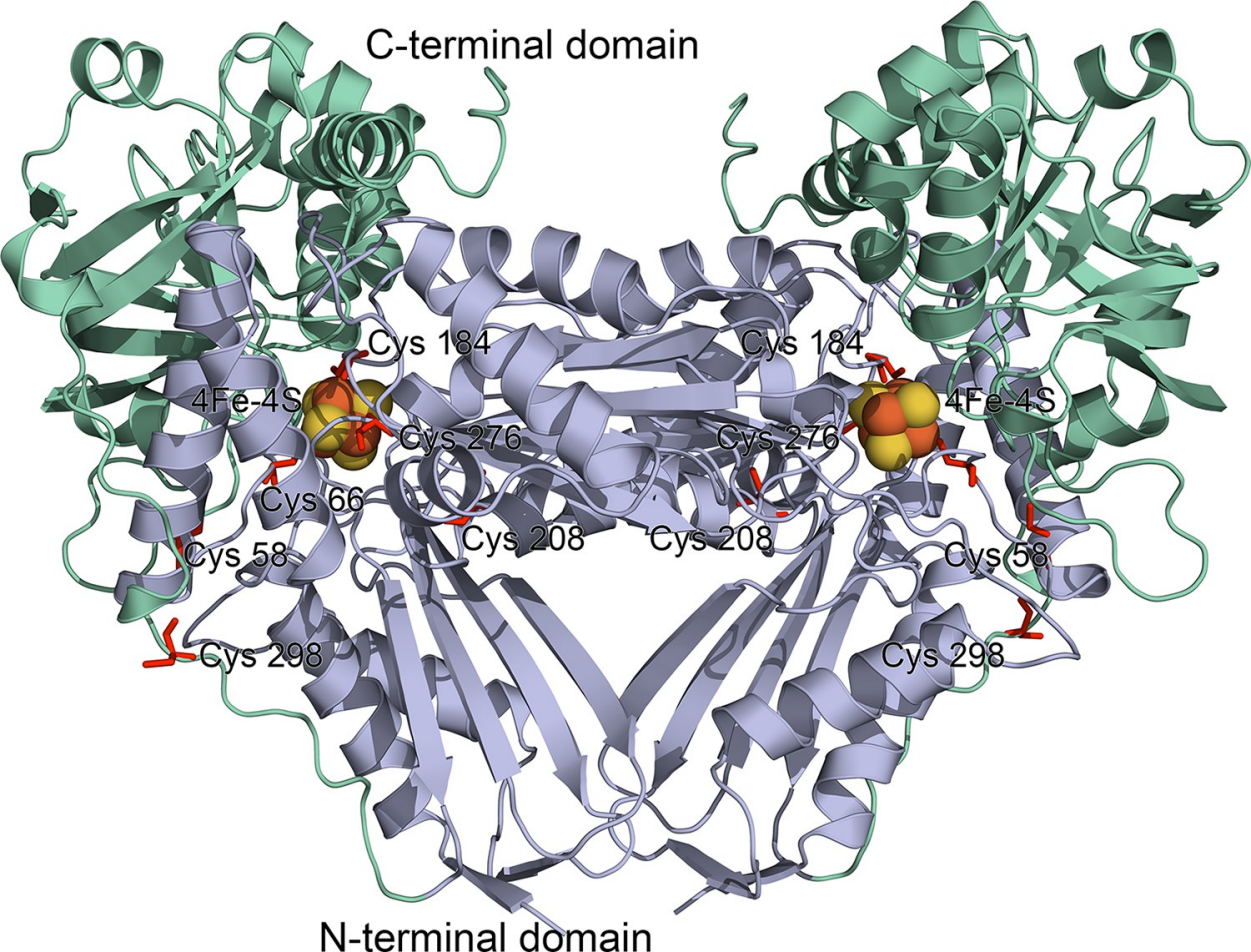
The model structure of FumI homodimer from *M*. *alcaliphilum* obtained by AlphFold [18]. The N-terminal (alpha-type catalytic) domain is light blue, C-terminal (beta-type catalytic) domain is light green. The iron–sulfur cluster was determined according to the structure of fumarase 2 from *Leishmania major* (PDB ID 5L2R). Cys residues are red.

The sequence of the class I fumarase from *M*. *alcaliphilum* (UniProt ID G4T1T1) corresponds to a protein with a length of 507 a.a., with a molecular weight of approximately 55 kDa; the isoelectric point is 5.51. PONDR v. VLXT predicts the protein to be 30% disordered. Each homodimer subunit has 6 cysteine residues. The predicted model (AlpfaFold2) of fumarase is represented as an asymmetric homodimer, with each monomer consisting of two structural domains arranged around an iron–sulfur catalytic cluster (Fig 4). Although there was only 23% identity of the translated amino acid sequences of the *L*. *major* and *M*. *alcaliphilum* is fumarases, the folding of the methanotrophic protein sequence showed the identity of the three-dimensional structure of these two fumarases (PDB ID 5L2R). The previously defined active site of *L*. *major* fumarase [22] includes [4Fe-4S] cluster and the following 12 residues from chain A: Cys-133, Gln-134, Asp-135, Arg-173, Gly-216, Cys-252, Cys-346, Arg-421, Thr-467, Thr-468, Arg-471, Lys-491, and His-334 from chain B. The alignment of amino acid sequences showed that the active site of *M*. *alcaliphilum* FumI was similar to that of *L*. *major*, except that Arg-173 is replaced by Leu (S3 Fig).

The monomer of the studied FumI from *M*. *alcaliphilum* has an iron–sulfur cluster coordinated by three cysteine residues C66, C184, and C276; the motif is C-X118-C-X92-C from the N-terminal domain. According to our fumarase model, there are only 2 cysteines on the surface of the molecule; so, it can be assumed that the protein will be less sensitive to oxygen than in some other microbes [23].

### The kinetic properties of *M*. *alcaliphilum* FumC

The FumC from *M*. *alcaliphilum* is active within a narrow pH range (Fig 3A, 3B). The enzyme demonstrated the maximum activity in the fumarate hydration reaction at pH 8.0 and in the malate dehydration reaction at pH 8.5. The optimum temperature for the reaction was 50°C (Fig 3C). FumC was a thermolabile enzyme. It could not withstand the exposure at 40°C, losing 80% activity after 30 minutes, and was completely inactivated after 10-min heating at 50°C. The enzyme activity was not changed after one-hour incubation at 30°C. In contrast, other class II fumarases are known as thermostable enzymes [7, 24, 25].

Unlike FumI, the *M*. *alcaliphilum* FumC has almost the same activity with fumarate or malate as a substrate (45 ± 1 and 42 ± 3 U mg^-1^, respectively). The forward reaction of FumC obeys the Michaelis–Menten kinetic equation, but the *S*_0.5_ value with Hill’s coefficient was used for the reverse reaction (S2B, S2D Fig). The *K*_m_ value for fumarate was 0.10 ± 0.001 mM, the *S*_0.5_ value for malate was 0.14 ± 0.01 mM, with the Hill’s coefficient *n* = 2.1 ± 0.4. FumC does not use mesaconate as a substrate, which is consistent with the results reported for the previously characterized class II fumarases. The specificities of FumC to both substrates were approximately the same (Table 1). Phosphoenolpyruvate and citrate reduced the activity of FumC by 30 and 50%, respectively (S2 Table).

### Phenotypic characterization of the mutant strains

Crude extracts obtained from the cells of *M*. *alcaliphilum* 20Z grown under methane or methanol showed a fumarase activity of 13 ± 0.7 or 28 ± 1.2 nmol min^-1^mg^-1^, respectively. The activity of FumI in the mutant strain *ΔfumC*Δ*mae* grown on either methane or methanol was found to be 27 ± 1.2 nmol min^-1^mg^-1^. The FumC activity in the *ΔfumI*Δ*mae* strain was approximately 3 ± 0.1 nmol min^-1^mg^-1^ regardless of the growth substrate. In the 20Z-3E strain (*ΔfumCΔfumIΔmae* genotype), fumarase activity was not found.

In the presence of methane, *M*. *alcaliphilum* 20Z-3E had a prolonged lag-phase and a half times slower growth rate (S4A Fig). In the presence of methanol, the triple mutant also had a lag-phase (S4B Fig).

**Fig 5:**
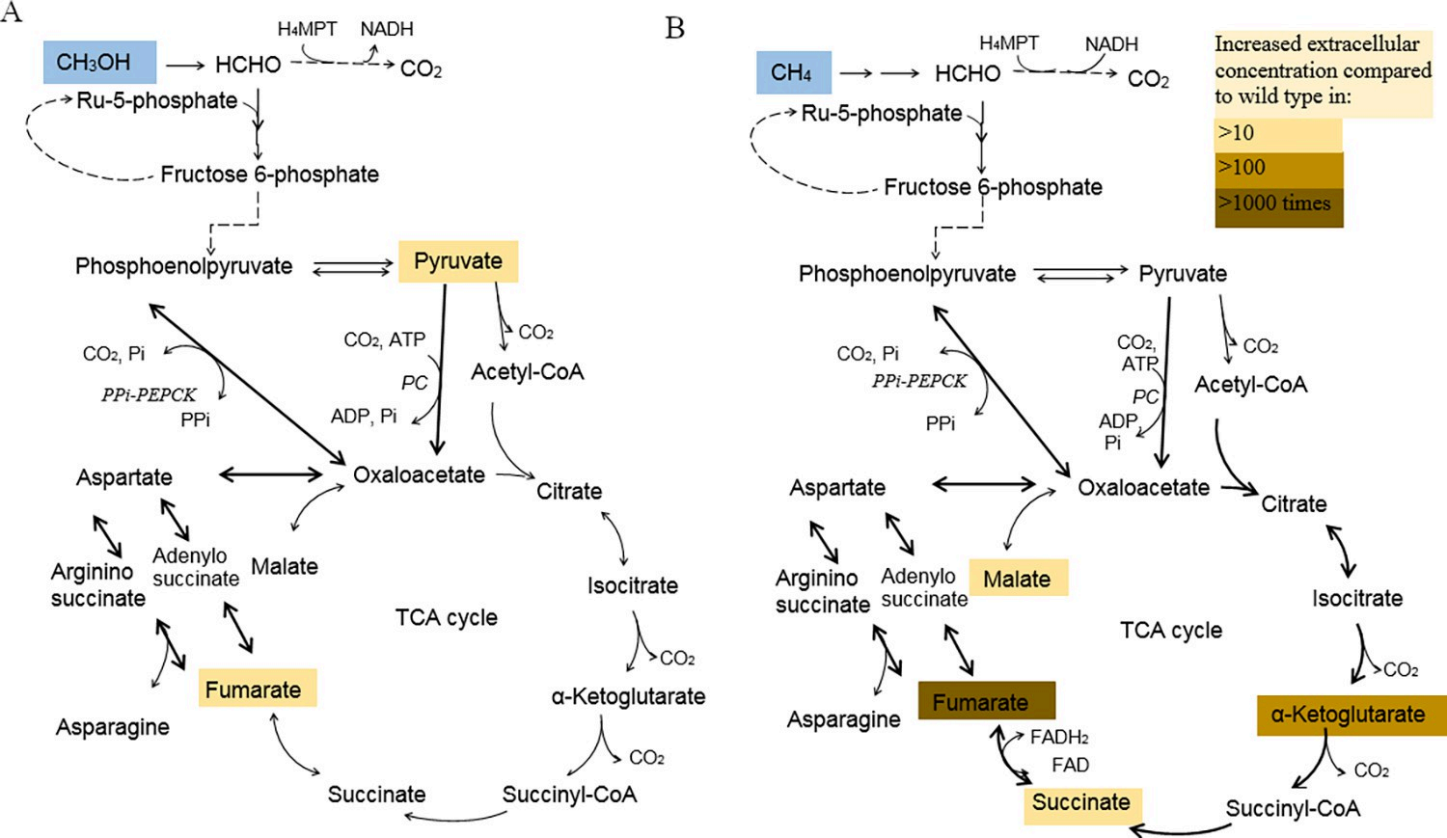
An increase in the concentration of extracellular metabolites in the triple mutant strain 20Z-3E compared to the wild type during the growth on methanol (A) and methane (B). PC–pyruvate carboxylase, PPi-PEPCK–PPi-dependent phosphoenolpyruvate carboxykinase.

The culture liquid of the *M*. *alcaliphilum* 20Z-3E grown under methane contained the increased concentrations of the TCA cycle intermediates as compared to the wild type: fumarate ~ 5000-fold, α-ketoglutarate ~ 200-fold, malate ~58-fold and succinate ~30-fold (Table 2, Fig 5). In case of growth on methanol, the 20Z-3E strain also accumulated the increased levels of the TCA cycle metabolites but to a much lesser extent: fumarate concentration was 50 times higher compared to WT cells (1150 ± 42 vs 18 ± 1 μmol g^-1^ DCW at WT; Table 2, Fig 5). Interestingly, the changes in the concentration of the Krebs cycle intermediates were not significant in the double mutants (Table 2).

**Table 2:** Analysis of the culture fluid of the mutant and WT strains of *M*. *alcaliphilum* growing on methane (μmol g^-1^ DCW).

Strain genotype	Formate	Fumarate	Succinate	Pyruvate	Malate	Citrate	α-Ketoglutarate
WT							
methane	72 ± 40	0.47 ± 0.06	3.2 ± 0.9	0.8 ± 0.6	5.6 ± 3.5	<0.01	0.68 ± 0.07
methanol	7.5 ± 0.8*	18 ± 1	19 ± 2	1.7 ± 0.6	114 ± 6	11.1 ± 0.5	9 ± 1
ΔfumIΔmae							
methane	68 ± 23	0.54 ± 0.03	2.4 ± 0.1	0.34 ± 0.23	1.9 ± 1.0	<0.01	0.3 ± 0.1
methanol	29 ± 4*	22 ± 2	16 ± 2	0.68 ± 0.03	42 ± 7	10.2 ± 1.5	24 ± 2
ΔfumCΔmae							
methane	42 ± 4	2.46 ± 0.50	2.0 ± 0.3	0.5 ± 0.2	0.39 ± 0.23	<0.01	0.06 ± 0.04
methanol	32 ± 7*	15.0 ± 0.7	27 ± 3	1.3 ± 0.7	72 ± 15	14.7 ±3.0	36 ± 2
20Z-3E (ΔfumIΔfumCΔmae)							
methane	90 ± 28	2647 ± 179	88.8 ± 10.5	1.9 ± 0.4	92 ± 47	<0.01	193 ± 30
methanol	15 ± 1*	1 150 ± 42	81 ± 6	56 ± 5	212 ± 25	8.3 ± 1.7	60 ± 3

*mmol g^-1^ DCW

### Distribution and phylogenetic analysis of fumarase in methanotrophs

**Fig 6:**
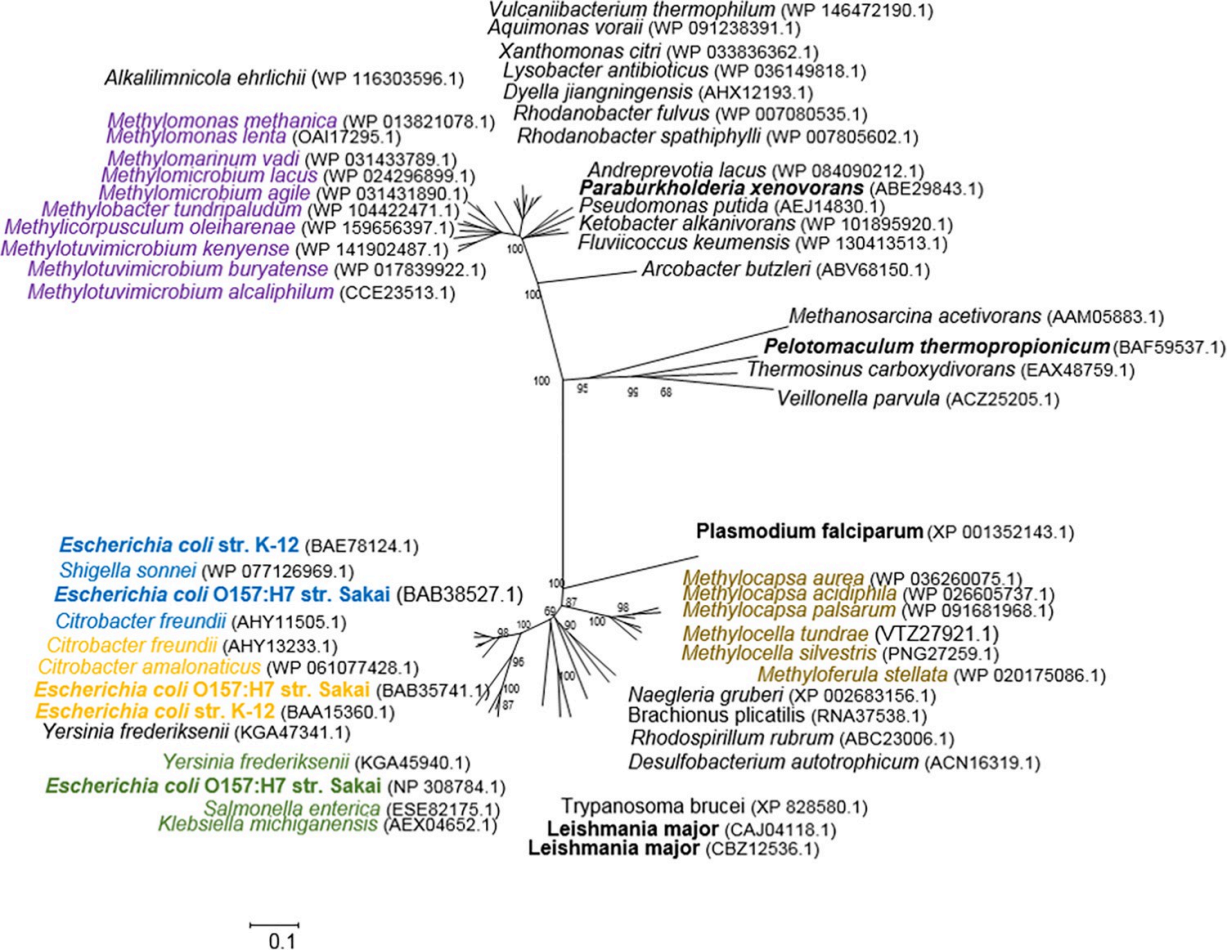
Phylogenetic tree of class I fumarases. Tree topography and evolutionary distances are given by the neighbor-joining method with Poisson correction. The characterized enzymes are in bold [1, 2, 5, 15, 26, 27]. The amino acid accession numbers in the GeneBank are in brackets. Alphaproteobacterial methanotrophs are brown, gammaproteobacterial methanotrophs are violet, bacteria with FumA are orange, bacteria with FumB are blue, and bacteria with FumD are green.

*M*. *alcaliphilum* 20Z possesses two fumarases, which are representatives of two non-homologous classes I and II (Fig 6, S5 Fig). As previously presented on the phylogenetic tree [2] fumarases, mesaconases and (2R,3R)-tartrate dehydratase are related enzymes. The fumarase of the methanotroph belonging to class I (accession number CCE23513.1) has only a 25% identity of translated amino acid sequences with the previously studied fumarases A and B from *E*. *coli* [4] and a 34% identity with the heterodimeric fumarase from *Pelotomaculum thermopropionicum* [1] (Fig 6, S4 Table). Although these enzymes belong to class I fumarases [2], the *fumI* gene of the methanotroph cannot be named as *fumA* by analogy with the *E*. *coli* gene.

FumI from gammaproteobacterial methanotrophs exhibits a high homology (70% identity) with the previously characterized enzyme from (*Para*)*burkholderia xenovorans*, which has higher specificity for mesaconate than for fumarate and functions more likely as a mesaconase [15]. Among alphaproteobacterial methanotrophs, only representatives of the family *Beijerinckiaceae* possess fumarases of class I, but it is absent in methanotrophs of the family *Methylocystaceae* (Fig 6, S3 Table). Fumarases from alphaproteobacterial methanotrophs have only a 25% identity with the *M*. *alcaliphilum* enzyme.

FumC belonging to class II is found in almost all methanotrophic bacteria with the exceptions *Methylomarinum vadi* and some representatives of the genus *Methylomonas* (S3 Table, S5 Fig). *M*. *alcaliphilum* FumC shows a 76% identity with the enzymes from *Methylobacter tundripaludum* and some members of the genus *Methylomonas* and about 57% identity with FumC from *E*. *coli* and the enzyme from Homo sapiens. Interestingly, the enzyme from *M*. *alcaliphilum* 20Z has ~ 62% identity with the class II fumarase from alphaproteobacterial methanotrophs.

Phylogenetic analysis has shown that the most of class II fumarases of gammaproteobacterial methanotrophs form a separate branch on the phylogenetic tree having only a 43% identity to FumC from *M*. *alcaliphilum* 20Z (S5 Fig). This group does not contain the previously characterized enzymes.

## Discussion

In this study, we have characterized for the first time two isoforms of the fumarase from an obligate methanotroph. The class II fumarase FumC from *M*. *alcaliphilum* 20Z is the more common form of the enzyme among gammaproteobacterial methanotrophs, though it is absent in some species of *Methylomonas* and *Methylomarinum* (S3 Table). Similarly to fumarases of this class, *M*. *alcaliphilum* FumC is a tetramer with a subunit molecular weight of ~50 kDa. Unlike *E*. *coli* FumC, which is more active in the reaction of fumarate hydration and more specific for fumarate than for malate [2], the enzyme from *M*. *alcaliphilum* 20Z exhibits equal activities in both reactions (~45 U mg^-1^) and similar substrate specificities (Table 1). A distinctive property of the *M*. *alcaliphilum* enzyme from *E coli* FumC is its thermolability. Interestingly, that fumarases from *Streptomyces lividans* and *S*. *thermovulgaris* maintained activity after incubation at 40°C within 48 and 24 h, respectively, whereas the enzyme *S*. *coelicolor* rapidly denatured at 50°C [24, 25].

Class I fumarase is found in a limited number of gammaproteobacterial methanotrophs (S3 Table, Fig 6). The methanotrophic class I fumarases form a separate branch on the respective phylogenetic tree, sharing only a 29% identity with the A and B isozymes from *E*. *coli*. The enzyme from *M*. *alcaliphilum* has properties different from those of other class I fumarases: it is relatively thermostable and insensitive to oxygen. The insensitivity of fumarase to oxygen correlates with the physiology of methanotrophs due to strict oxygen dependence of methane oxidation. It has been shown that the operation of three *E*. *coli* class I isoenzymes was determined by cell growth conditions: the main enzyme was FumA under microoxic conditions, FumB under anoxic conditions, and FumC under oxygen conditions [6]. In *L*. *major* that can grow at various oxygen levels [28], two isoforms of class I fumarases had low *K*_m_ to substrate under aerobic conditions but higher activity under anaerobic conditions (S4 Table) [26].

As is known, the most of [Fe-S] proteins wrap their clusters inside the polypeptide, preventing reactive oxygen species (ROS) molecules from contacting the clusters and thereby shielding the clusters from damage [3, 23]. Oxidation of dehydratases for a long time can damage the clusters; sometimes they degrade beyond the [3Fe-4S]^+^ state, forming [2Fe-2S] clusters or even iron-free apoproteins. In most [4Fe-4S] dehydratases, three of the four iron atoms in the cluster are bound to the protein skeleton through the thiol groups of cysteines; one iron atom is solvent-exposed and coordinates the substrate [29]. The three cysteine residues that bind the non-catalytic iron atoms form a specific structural motif “C-Xn-C-X2-C". Based on *M*. *alcaliphilum* FumI model, there are only 2 cysteines on the surface of the molecule; so, it can be assumed that the protein will be less sensitive to oxygen than in some other microbes [23].

The *M*. *alcaliphilum* FumI is two-fold more active in the reaction of fumarate hydration compared to malate dehydration and is more specific to fumarate than to malate (Table 1). The *M*. *alcaliphilum* FumI also effectively reacts with mesaconate (methylfumarate) forming S-citramalate. Unlike *Burkholderia xenovorans*, *M*. *alcaliphilum*, being an obligate methanotroph, cannot use C5 dicarboxylic acids such as mesaconate or itaconate as a carbon source. In bacteria of the family *Enterobacteriaceae*, the source of mesaconate can be the sequential transformation of glutamate by glutamate mutase and 3-methylaspartase [30, 31]. For *Aspergillus niger* and *Alcaligenes xylosoxydans*, it was shown that the source of C-citramalate is itaconate, which, in the case of eukaryotes, can be formed from *cis*-aconitate by aconitate decarboxylase [32, 33]. It has been also shown that in macrophages, mesaconate is synthesized endogenously from itaconate [34]. However, these enzymes participating in the interconversion of C5-compounds are not encoded by the genome of the methanotroph. Nonetheless, *M*. *alcaliphilum* 20Z has the citM gene, which encodes citramalate synthase (2.3.3.21; MALCv4_1503) catalyzing the formation of (R)-citramalate from pyruvate and acetyl-CoA in the threonine-independent pathway of isoleucine biosynthesis (S6 Fig) [35]. Although there is some correlation between the presence of FumI and the *citM* and *leuB* genes in the gammaproteobacterial methanotrophs (S3 Table), there is no direct evidence for the involvement of FumI in the isoleucine synthesis. The use of mesaconate as the substrate is a common property of class I fumarases [2], but its physiological significance for the methanotroph is unclear.

Fumarase is one of the Krebs cycle enzymes, which has an energy function in aerobic bacteria. Methanotrophs generate the main energy oxidizing C1 substrate to CO_2_ via methanol, formaldehyde and formate (Fig 1). The oxidative TCA cycle operates at methane growth but not methanol growing culture [10, 11]. As shown by fumarase gene knockout, the class I fumarase in *M*. *buryatense* 5GB1 is the main form of the enzyme [10]. On the contrary, our results demonstrate that in *M*. *alcaliphilum* 20Z these fumarases are interchangeable, since the activity of FumC was detectable in the extracts of the *ΔfumIΔmae* strain cells as well as the activity of FumI was measurable in the extracts from the *ΔfumCΔmae* cells. In addition, turning off each of the genes did not lead to physiological differences from the wild type (S4 Fig, Table 2). Both enzymes are functional in the halotolerant *M*. *buryatense* 5GB1, where class I fumarase plays a key role [10]. Most other methanotrophs have only FumC and lack FumI (S4 Table). The absence of fumarate in the culture liquid of mutant strains with the *ΔfumIΔmae* and *ΔfumCΔmae* genotypes confirms interchangeability of the two forms of fumarases; therefore, the Krebs cycle continues to function. The accumulation of fumarate in the triple mutant growing on methane indicates that the Krebs cycle continues to function, replenishing the oxaloacetate pool in anaplerotic reactions. There are two candidates for C3 carboxylation reactions: PPi-PEP-carboxykinase (PEPCK, CCE23879) or two-subunit biotin-dependent pyruvate carboxylase (PC, CCE24021/CCE24020) (Figs 1, 5). The methane growing strain accumulated higher level of fumarate than the methanol growing one. It is consistent with the previously proposed different functions of the TCA cycle on these substrates and its incompleteness during the growth on methanol [11, 36]. The absence of a significant increase in the extracellular pool of other intermediates of the TCA cycle also indirectly indicates the nonfunctionality of this cycle in methanol growing culture. Interestingly, oxaloacetate formed in anaplerotic reactions can be converted to fumarate bypassing malate via the adenylosuccinate pathway (Figs 1, 5). Additional shutdown of the gene encoding malic enzyme irreversibly decarboxylating malate to pyruvate [12] probably enhances the oxaloacetate flow into the TCA cycle or into the adenylosuccinate pathway.

The disruption of the gene encoding another TCA cycle enzyme, succinate dehydrogenase, yield in the type I methanotroph *Methylomonas* sp. DH-1 has led to the accumulation of succinate 95 mg L^-1^ with a productivity of 0.246 mmol g^-1^ DCW d^-1^ [37]. The yield of fumarate accumulating in *M*. *alcaliphilum* 20Z-3E (2.6 mmol g^-1^ DCW or 142 mg L^-1^) is approximately comparable. Thus, knockout of one stage of the TCA cycle does not have a significant effect on methanotrophic growth, but can lead to the production/accumulation of organic acids. *M*. *alcaliphilum* 20Z codes biotin-dependent pyruvate carboxylase whereas *Methylomonas* sp. DH-1 possesses PEP carboxylase, which can recover C4-intermediates of the TCA cycle (class I) (S4 Table).

## Conclusion

Bioinformatic (Genomic) analysis suggests that, despite the obligate dependence of methanotrophs on the C1 substrate, these bacteria demonstrate a quite flexible central metabolism, which obviously promotes adaptation to their natural econiche. The redundancy of central metabolic pathways makes methanotrophs attractive targets for their modification, including generation of the mutants producing the valuable metabolites without the loss of the survival potential. Fumarase is an essential enzyme in the TCA cycle, whose function in the methanotrophs obtaining energy for growth via C1 oxidation is mainly to supply amino acid precursors and other organic metabolites for biosynthesis. In *M*. *alcaliphilum* and many other gammaproteobacterial methanotrophs, there are two unrelated fumarases with different properties. The most pronounced features of the FumI from *M*. *alcaliphilum* are high activity and stability under aerobic conditions, which distinguishes (differentiates) it from the previously characterized class I fumarases from other bacteria and correlates with the strict aerobiosis of methanotrophs. The fumarate pool accumulated by the methanol growing triple mutant is lower compared to the methane-growing culture. It is in accordance with the previous finding that the TCA cycle in the methanol growing methanotroph *M*. *buryatense* is incomplete [11, 36]. If this is the case for *M*. *alcaliphilum*, the carbon flow via the oxidative TCA cycle is essential for this bacterium (for fumarate formation).

## Supporting information

S1 Table Primers used in the work. (PDF)

S2 Table Effect of metabolites on the *Mtm*. *alcaliphilum* fumarase activity. (PDF)

S3 Table Distribution of class I and II fumarases, as well as some enzymes of isoleucine biosynthesis among methanotrophs. The data were obtained from databases www.genoscope.cns.fr, http://www.ncbi.nlm.nih.gov/, and https://img.jgi.doe.gov.
(PDF)

S4 Table Kinetic parameters of some class I and II fumarases. (PDF)

S1 Fig Gel electrophoresis of PCR products obtained with the primers fumI-F and fumI-R for the *fumI* gene (A, line 1–5), fumI-F-up and fumI-down-R for the up-down fragment of the *fumI* gene (A, lines 6–10), fumC-F and fumC-R for the *fumC* gene (B, lines 1–6), fumC-F-up and fumC- down-R for the up-down fragment of the *fumC* gene (B, lines 6–10), Mae-Acc-Nde-F and Mae-Xho-R [11] for the *sfc* gene (C, lines 1–5), mae-F-up and mae-down-R for the up-down fragment of the *mae* gene (C, lines 6–10) using template DNA: from the *ΔmaeΔfumI* strain (lanes 2, 7), *ΔmaeΔfumIΔfumC* strain (lanes 3, 8), *ΔmaeΔfumC* strain (lanes 4, 9), the wild type strain (lane 5, 10). Lines 1 and 6 were negative control without DNA; M, molecular mass markers GeneRulerTM DNA Ladder Mix (Thermo Scientific).
(PDF)

S2 Fig Velocity versus substrate curves of the *M*. *alcaliphilum* FumI (A, C, E) and FumC (B, D) toward malate (A, B), fumarate (C, D), and mesaconate (E) as a substrate. *V*_max_ is the maximum velocity of the reaction, *[S]* is the substrate concentration, *K*_m_ is the concentration of the substrate when the reaction velocity is half of *V*_max_, *n* is Hill coefficient, *S*_0.5_ is the substrate concentration at half *V*_max_ when *n* ≠ 1.
(PDF)

S3 Fig Multiple alignment of the primary structure of class I fumarase: Mtm. alc (CCE23513.1), L.major (CBZ12536.1), P.therm (BAF59537.1), B.xenov (ABE29843.1), E.coli (BAE78124.1). The asterisk indicates amino acid residues forming active site.
(PDF)

S4 Fig The growth of *M*. *alcaliphilum* 20Z (red line), and mutant strains with genotype *ΔmaeΔfumIΔfumC* (green line), *ΔmaeΔfumI* (blue line) and *ΔmaeΔfumC* (yellow line) on methane (A) and methanol (B) in the presence of 3% NaCl.
(PDF)

S5 Fig Phylogenetic tree of class II fumarases. There were a total of 565 positions in the final dataset. Tree topography and evolutionary distances are given by the neighbor-joining method with Poisson correction. The scale bar represents a difference of 0.1 substitutions per site. The characterized enzymes are in bold [1, 8, 10, 12–15]. The amino acid accession numbers in the GeneBank are in brackets. Alpha-proteobacterial methanotrophs are brown, gamma-proteobacterial methanotrophs are violet.
(PDF)

S6 Fig Pathways for isoleucine biosynthesis in *M*. *alcaliphilum* 20Z [16, 17, KEGG Pathways from www.genoscope.cns.fr]. *citM*–citramalate synthase, *leuB*–isopropylmalate dehydrogenase, *leuCD*– 3-isopropylmalate dehydratase/isomerase.
(PDF)
