# Supplementary material for: Interchangeability of class I and II fumarases in an obligate methanotroph Methylotuvimicrobium alcaliphilum 20Z
Source: PLoS One. 2023 Oct 26;18(10):e0289976. doi: 10.1371/journal.pone.0289976 (PMC10602362; doi:10.1371/journal.pone.0289976)
Supplement: S1 Table — (PDF) [file pone.0289976.s001.pdf]

S1 Table. Primers used in the work

Primer	Sequence (5'-3 ') with indication of the restriction endonucleases	Target
fumI-F	ACCATATGACCTTGATTCGCCAAGAAGACT (NdeI)	*fumI* gene
fumI-R	ATGAGCTCACTTTCGAAACCACCGGAATTTTGCCT (SacI)	
fumI-F-up	ATAGATCTATCAACTGAGTTGTACGCGG (BglII)	The upper flanking region of the *fumI* gene
fumI-R-up	ACCATATGACGCCGTGAACCCAGCACCTAAATTA (NdeI)	
fumI-F-dawn	ACGGGCCCCTAACGGTCATGAAAGCCTG (ApaI)	The lower flanking region of the *fumI* gene
fumI-dawn-R	AAGAGCTCCAATCGAGTCAACGTAAACCAAT (SacI)	
fumC-F	ATCATATGACAATCGGAAGTTCGGCC (NdeI)	*fumC* gene
fumC-R	ACCTCGAGTTTCGGCATGATCATCCGTTCC (XhoI)	
fumC-F-up	ACAGATCTACGTTTTGACTTTGGCCGAA (BglII)	The upper flanking region of the *fumC* gene
fumC-up-R	ATGGTACCGATGGATTATTCGCTTGGTT (Acc65I)	
fumC-F-dawn	ATGGGCCCATGCACGTAAGCTATGAACA (ApaI)	The lower flanking region of the *fumC* gene
fumC-dawn-R	ACGAGCTCAACCCATGATAAAAAGCGAT (SacI)	
mae-F-up	GAC**AGATCT**CAGGGATTGCCTCGAAACA (BglII)	The upper flanking region of the *mae* gene
mae-up-R	CAG**CATATG**ACGGAAGTTTCCTTAGTCAACCT (NdeI)	
mae-F-dawn	TATGGGCCCCGTATAGCATCCGATGGGTG (ApaI)	The lower flanking region of the *mae* gene
mae-dawn-R	AAGGAGCTCGCCTTGGCTGCTTATCTCGG (SacI)	
