# Supplementary material for: Interchangeability of class I and II fumarases in an obligate methanotroph Methylotuvimicrobium alcaliphilum 20Z
Source: PLoS One. 2023 Oct 26;18(10):e0289976. doi: 10.1371/journal.pone.0289976 (PMC10602362; doi:10.1371/journal.pone.0289976)
Supplement: S2 Table — (PDF) [file pone.0289976.s002.pdf]

S2 Table. Effect of metabolites on the *Mtm. alcaliphilum* fumarase activity.

Metabolites	Concentration, mM	Related activity, %	
		Class I fumarase	Fumarase C
Control	-	100	100
Phosphoenolpyruvate	1	87 ± 3	**74 ± 2**
Glucose-6-phosphate	5	87 ± 3	89 ± 2
Fructose-6-phosphate	5	89 ± 2	112 ± 2
Fructose-1,6-bisphosphate	5	114 ± 3	111 ± 3
Oxaloacetate	1	91 ± 2	103 ± 3
Citrate	1	92 ± 3	**55 ± 2**
PPi	1	94 ± 3	114 ± 4
Lactate	1	105 ± 2	100 ± 2
α-ketoglutarate	1	114 ± 3	110 ± 3
Succinate	1	**144 ± 5**	95 ± 3
Serine	1	117 ± 4	104 ± 1
Hydroxypyruvate	1	117 ± 3	116 ± 2
Glycine	1	127 ± 2	**141 ± 4**
Pyruvate	1	135 ± 3	123 ± 3
