# Supplementary material for: Interchangeability of class I and II fumarases in an obligate methanotroph Methylotuvimicrobium alcaliphilum 20Z
Source: PLoS One. 2023 Oct 26;18(10):e0289976. doi: 10.1371/journal.pone.0289976 (PMC10602362; doi:10.1371/journal.pone.0289976)
Supplement: S3 Table — The data were obtained from databases www.genoscope.cns.fr, http://www.ncbi.nlm.nih.gov/, and https://img.jgi.doe.gov. (PDF) [file pone.0289976.s003.pdf]

S3 Table. Distribution of class I and II fumarases, as well as some enzymes of isoleucine biosynthesis among methanotrophs. The data were obtained from databases www.genoscope.cns.fr, http://www.ncbi.nlm.nih.gov/, and https://img.jgi.doe.gov.

**Bacteria**	*fumI*	*citM*	*leuB*	*leuD/C*	*fumII(fumC)*
Gammaproteobacteria					
Methylotuvimicrobium alcaliphilum 20Z	MALCv4_1827	MALCv4_1503	MALCv4_1502	MALCv4_3528, MALCv4_3529	MALCv4_0281
Methylotuvimicrobium buryatense 5G	MBURv2_60244	MBURv2_30147	MBURv2_30146	MBURv2_210153 MBURv2_210154	MBURv2_130865
Methylotuvimicrobium kenyense AMO1	2590636623	2590633224	MKEN_v1_10274	2590634658/ 2590634657	2590634930
Methylobacter tundripaludum SV96	648843431	648843297	648841877	648843299/ 648843298	648841651
Methylobacter tundripaludum 21/22	2563533305	2563530179	2563532103	2563530176/ 2563530177	2563533974
Methylobacter tundripaludum 31/32	2574197939	2574194697	2574196904	2574194695/ 2574194696	2574194047
Methylobacter tundripaludum-129 (UID203)	2634846886	2634847169	2634846554	2634847172/ 2634847171	2634848533
Methylobacter tundripaludum OWC-DMM	2706256061	2706257309	2706255665	2706257307/ 2706257308	2706254956
Methylobacter tundripaludum OWC-G53F	2706250150	2706251356	2706250924	2706251354/ 2706251355	2706252630

Methylobacter oryzae KRF1	2881007815	2881005603	2881005947	2881005607/ 2881005606	2881006797
Methylicorpusculum oleiharenae XLMV4	2522718734	2522716610	2522716609	2522718043/ 2522718044	2522719860
Methylomicrobium agile ATCC 35068	2574335353	2574332302	2574333405	2574335526/ 2574335527	2574334228
Methylomicrobium album BG8	2508548776	2508549762	2508546872	2508548988/ 2508548987	2508547683
Methylomonas sp. FA2	2974475065	2974475970	2974475974/ 2974476380	2974476378/ 2974476379	+? incorrect sequence
Methylosarcina fibrata AML-C10	2517554377	MFIBv2_51492	2517556760	2517555755/ 2517555754	2517553601
Methylosarcina lacus LW14	2517247019	MLACv1_3283	2517249934	2517246713/ 2517246714	2517246970
Methylomonas lenta R-45370	2745458078	2745456622	2745456620	2745456624/ 2745456623	2745459303
Methylomonas denitrificans FJG1	2775380365	2775378934	2745442456/ 2775379925	2775379923/ 2775379924	2775378859, 2775378341
Methylomonas methanica NCIMB 11130	2745444195	2745441884	2745442456/ 2745442841	2745442843/ 2745442842	2745442421
Methylomonas methanica S-1	2784216483	2784215817	2784216817/ 2784214634	2784214632/ 2784214633	2784217485

Methylomonas methanica R-45363	2745451222	2745450121	2745450127/ 2745450400	2745450398/ 2745450399	2745451096
Methylomonas methanica R-45371	2745453944	2745454673	2745454667/ 2745454820	2745454818/ 2745454819	2745452178
Methylomarinum vadi	2574470425	2574469459	2574469457	2574470172/ 2574470171	-
Methylomonas koyamae JCM 16701	2731416215	2731416291	2731416301/ 2731413890	2731413893/ 2731413892	-
Methylomonas sp. LW13	2561704851	2561706102	2561706107/ 2561707413	2561707415/ 2561707414	-
Methylomonas sp. Kb3	2894514104	2894511974	2894511969/ 2894511515	2894511517/ 2894511516	-
Methylomonas sp. ZR1	2911043546	2911040417	2911040414/ 2911044243	2911044245/ 2911044244	-
Methylomonas sp. DH-1	2841135521	2841136704	2841136717/ 2841135875	2841135877/ 2841135876	-
Methylomonas koyamae R-45378	2838829805	2838831549	2838831703/ 2838832138	2838832140/ 2838832139	-
Methylomonas koyamae R-45383	2745462831	-	2745465428	2745465426/ 2745465427	-
Methylomonas koyamae R-49807	2745467715	2745468987	2745470272/ 2745467167	2745467169/ 2745467168	-

Methylomonas koyamae LM6	2812977174	2812978317	2812978324/ 2812977514	2812977516/ 2812977515	-
Methylomonas rhizoryzae GJ1	2989396061	2989392591	2989392592/ 2989393255	2989393257/ 2989393256	-
Methylomonas methanica MC09	2504963263	2504962289	2504962290/ 2504963773	2504963775/ 2504963774	-
Methylomonas sp. 11b	2516191377	2516189360	2516189364/ 2516192346	2516192348/ 2516192347	-
Methylomonas sp. MK1	2522500200	2522497380	2522497385/ 2522499235	2522499233/ 2522499234	-
Methylobacter whittenburyi ACM 3310	-	-	2585414544	2585414542/ 2585414543	2585414359
Methylocaldum szegediense O-12	-	-	2508834529	2508834531/ 2508834530	2508830494
Methylocaldum marinum S8	-	-	2832928730	2832928732/ 2832928731	2832923498
Methylogaea oryzae JCM 16910	-	-	2677969858	2677967552/ 2677969856	2677966979
Methyloglobulus morosus KoM1	-	2528840112	2528839063	2528841232/ 2528841233	2528840190
Methylococcus capsulatus Bath	-	-	637171217	637171219/ 637171218	637170716

Methylococcus capsulatus Texas	-	-	2547694157	2547694159/ 2547694158	2547693297
Methylohalobius crimeensis 10Ki	-	2525294777	2525294857	2525294855/ 2525294856	2525296527
Methylomagnum ishizawai 175	-	-	2595104464	2595104462/ 2595104463	2595103871
Methylospira mobilis Shm1	-	-	2883190278	2883190276/ 2883190277	2883189294
Methylovulum miyakonense HT12	-	-	2516961228	2516961226/ 2516961227	2516959769
Methylovulum psychrotolerans Sph1	-	-	2833294236	2833294233/ 2833294234	2833292666
Alphaproteobacteria					
Methylocapsa aurea KYG T	2576793726	-	2576792227	2576791774/ 2576794806	2576791617
Methylocapsa acidiphila B2	2510253629	-	2510255703	2510254980/ 2510256919	2510254497
Methylocapsa palsarum NE2	2676341146	-	2676342005	2676340455/ 2676340913	2676339496
Methyloferula stellata AR4	2516990424	-	2516989751	2516990152/ 2516989324	2516989028
Methylocella silvestris BL2	643463496	-	643466801	643463971/	643463616

				643464488	
Methylocella silvestris TVC	2831734345	-	2831733217	2831731970/ 2831732070	2831735218
Methylocella tundrae T4	VTZ27921.1	VTZ27780.1	2836739001	VTZ25629.1 / VTZ28168.1	VTZ27404.1
Methylocystis heyeri H2	-	2995291347			2995291027
Methylocystis parvus OBBP	-	2549983782			2549981944
Methylocystis rosea SV97T	-	2517401853			2517401733
Methylocystis silviterrae FS	-	2910664575			2910664443
Methylosinus trichosporium OB3b	-		2805051882	2805051296/ 2805050769	2805050760
Methylosinus sporium DSM 17706	-		2839753716	2839754888/ 2839753955	2839753945
Verrucomicrobia					
Methylacidiphilum kamchatkense Kam1	-		2630033810	2630033859/ 2630033811	fragment
Methylacidiphilum kamchatkense Kam1	-		2771470594	/ 2771470593	2771469846
Methylacidiphilum infernorum V4	-		642665797	642664955/ 642665796	642665005
Methylacidiphilum fumariolicum SolV	-		2539612595	2539612506/ 2539612594	2539612465

Methylacidiphilum fumariolicum Fur	-		2890952623	2890953673/ 2890952624	2890951755
Methylacidiphilum fumariolicum Ice	-		2890971145	2890971813/ 2890971146	2890971881
Methylacidiphilum fumariolicum Fdl	-		2891036944	2891037352/ 2891036943	2891036802
Methylacidiphilum fumariolicum Rib	-		2891042657	2891041917/ 2891042656	2891042509
Methylacidimicrobium tartarophylax 4A	-		2886600187	2886601595/ 2886600186	2886600084

fumI – fumarase I class, *citM* – citramalate synthase, *leuB* – isopropylmalate dehydrogenase, *leuCD* – 3-isopropylmalate dehydratase/isomerase, *fumC* – fumarase II class. The methanotrophs having both form fumarase are violet, the methanotrophs with only fum I are orange, the methanotrophs with only fumarase II class (or fumC) methanotrophs are green. The amino acid sequences of the isopropylmalate dehydrogenase (leuB) highlighted in black and red show approximately 20% identity.
