# Supplementary material for: Interchangeability of class I and II fumarases in an obligate methanotroph Methylotuvimicrobium alcaliphilum 20Z
Source: PLoS One. 2023 Oct 26;18(10):e0289976. doi: 10.1371/journal.pone.0289976 (PMC10602362; doi:10.1371/journal.pone.0289976)
Supplement: S4 Table — (PDF) [file pone.0289976.s004.pdf]

S4 Table. Kinetic parameters of some class I and II fumarases.

	Subunit molecular mass, kDa	V_{max}, U mg^{-1}			K_m ($S_{0.5}$^{a}), mM			k_{cat}/K_m($S_{0.5}$^{a}), s^{-1} M^{-1}			% identity [e]	
		Fumarate	Mesaconate	Malate	Fumarate	Mesaconate	Malate	Fumarate	Mesaconate	Malate		
Fumarase I class:												
M. alcaliphilum[c]	60	94	81	50	0.28 [a]	0.24[a]	0.55[a]	7.1×10^2	7.5×10^2	1.8×10^2		This study
Burkholderia xenovorans[d]	60	296	117	118	0.1	0.03	0.28	2.8×10^6	3.6×10^6	3.98×10^5	70	[1]
Pyrococcus furiosus[d]	18 and 30	1376		1892	0.34		0.41	3.2×10^6		3.7×10^6	22	[2]
Pelotomaculum thermopropionicum[d]	30 and 34	219[b]		25.2[b]	0.43		0.59	0.5×10^6		4.3×10^4	34	[3]
Leishmania major (isoform 1)	60	26.4/ 1.8[c]		11.8[d]/ 0.5[c]	2.5/1.3[a]		2.3/ 1.2[c]	11×10^3 [d]/ 1.5×10^3 [c]		5.6×10^3 [d]/ 4.6×10^2 [c]	23	[4]
Leishmania major (isoform 2)	60	186.2[d] /44.3[c]		138.1[d]/22.7[c]	5.7/1.9[a]		12.6[d]/ 5.7[c]	35.8×10^3 [d]/ 25.6×10^3 [c]		12×10^3 [d]/ 4.4×10^3 [c]	24	[4]
Trypanosoma cruzi [d] (cytosolic isoform)	62	400		290	0.8[a]		2.5[a]	8.2×10^6		1.3×10^6	21	[5]
Trypanosoma cruzi [d] (mitochondrial isoform)	62	2300		1050	1.5		2.8	1.5×10^6		0.4×10^6	21	[5]
E. coli K-12 [d] (fumA)	60	614	55.6	350	0.094	0.22	0.4	6.6×10^6	2.5×10^5	8.7×10^5	25	[6]
E. coli K-12 [d] (fumB)	60	654	57.8	289	0.21	0.1	0.78	3.12×10^6	5.8×10^5	3.7×10^5	25	[6]

E. coli O157:H7 [d] (fumD)	60	671	292/38[a]	52	0.9	0.15	0.2	7.5×10^5	2×10^6	2.6×10^5	20	[6]
Plasmodium falciparum [d]	60	182[b]	60[b]	159[b]	2.6	3.2	1.2	7.0×10^4	1.9×10^4	1.3×10^5	16	[7]
Fumarase II class:												
M. alcaliphilum	50	45	<0.1	42	0.11		0.14[a]	1.2×10^3		1.0×10^3		This study
Burkholderia xenovorans	50	376	<0.1	165	0.138	-	0.42	2.2×10^6	-	3.2×10^5	56	[1]
E. coli K-12 (fumC)	50	1164	<0.1	431	0.28		0.93				55	[6]
Human	50	344.8	-	178.6	0.207	-	0.857	5.56×10^6	-	6.9×10^5	58	[8]
Thermus thermophilus	46	1300	-			-	1		-		52	[9]
Corynebacterium glutamicum	50	720/650[b]	-	48/290[b]	4.2/0.67	-	1.8/5		-		46	[10]

[a] It is value of $S_{0.5}$; [b]It is value of k_{cat}; [c]Aerobic purification protein, which be reactivated with Fe^{2+} and thiol; [d] Anaerobic purification protein;

[e] % identity of translated amino acid sequences previously studied fumarases with FumI or FumC *M. alcaliphilum*.

References

1. Kronen M, Sasikaran J, Berg IA. Mesaconase Activity of Class I Fumarase Contributes to Mesaconate Utilization by *Burkholderia xenovorans*. Appl Environ Microbiol. 2015, 81: 5632-8. doi: 10.1128/AEM.00822-15.
2. van Vugt-Lussenburg BMA, van der Weel L, Hagen WR, Hagedoorn PL. Identification of two [4Fe-4S]-cluster-containing hydro-lyases from Pyrococcus furiosus. Microbiology (Reading). 2009;155: 3015–3020. doi:10.1099/MIC.0.030320-0
3. Shimoyama T, Rajashekhara E, Ohmori D, Kosaka T, Watanabe K. MmcBC in *Pelotomaculum thermopropionicum* represents a novel group of prokaryotic fumarases. FEMS Microbiol Lett. 2007;270: 207–213. doi:10.1111/J.1574-6968.2007.00665.X

4. Feliciano PR, Gupta S, Dyszy F, Dias-Baruffi M, Costa-Filho AJ, Michels PAM, et al. Fumarate hydratase isoforms of *Leishmania major*: subcellular localization, structural and kinetic properties. Int J Biol Macromol. 2012;51: 25–31. doi:10.1016/J.IJBIOMAC.2012.04.025
5. de Pádua RAP, Kia AM, Costa-Filho AJ, Wilkinson SR, Nonato MC. Characterisation of the fumarate hydratase repertoire in *Trypanosoma cruzi*. Int J Biol Macromol. 2017 Sep;102:42-51. doi: 10.1016/j.ijbiomac.2017.03.099
6. Kronen M, Berg IA. Mesaconase/Fumarase FumD in Escherichia coli O157:H7 and Promiscuity of *Escherichia coli* Class I Fumarases FumA and FumB. PLoS One. 2015;10. doi:10.1371/JOURNAL.PONE.0145098
7. Jayaraman V, Suryavanshi A, Kalale P, Kunala J, Balaram H. Biochemical characterization and essentiality of *Plasmodium fumarate* hydratase. Journal of Biological Chemistry. 2018;293: 5878–5894. doi:10.1074/jbc.M117.816298
8. Estévez M, Skarda J, Spencer J, Banaszak L, Weaver TM. X-ray crystallographic and kinetic correlation of a clinically observed human fumarase mutation. Protein Sci. 2002 Jun;11(6):1552-7. doi: 10.1110/ps.0201502. PMID: 12021453
9. Mizobata T, Fujioka T, Yamasaki F, Hidaka M, Nagai J, Kawata Y. Purification and characterization of a thermostable class II fumarase from *Thermus thermophilus*. Arch Biochem Biophys. 1998 Jul 1;355(1):49-55. doi: 10.1006/abbi.1998.0693.
10. Genda T, Watabe S, Ozaki H (2006) Purification and Characterization of Fumarase from *Corynebacterium glutamicum*, Bioscience, Biotechnology, and Biochemistry, 70:5, 1102-1109, DOI: 10.1271/bbb.70.1102
