# Supplementary material for: Interchangeability of class I and II fumarases in an obligate methanotroph Methylotuvimicrobium alcaliphilum 20Z
Source: PLoS One. 2023 Oct 26;18(10):e0289976. doi: 10.1371/journal.pone.0289976 (PMC10602362; doi:10.1371/journal.pone.0289976)
Supplement: S1 Fig — Gel electrophoresis of PCR products obtained with the primers fumI-F and fumI-R for the fumI gene (A, line 1–5), fumI-F-up and fumI-down-R for the up-down fragment of the fumI gene (A, lines 6–10), fumC-F and fumC-R for the fumC gene (B, lines 1–6), fumC-F-up and fumC- down-R for the up-down fragment of the fumC gene (B, lines 6–10), Mae-Acc-Nde-F and Mae-Xho-R [11] for the sfc gene (C, lines 1–5), mae-F-up and mae-down-R for the up-down fragment of the mae gene (C, lines 6–10) using template DNA: from the ΔmaeΔfumI strain (lanes 2, 7), ΔmaeΔfumIΔfumC strain (lanes 3, 8), ΔmaeΔfumC strain (lanes 4, 9), the wild type strain (lane 5, 10). Lines 1 and 6 were negative control without DNA; M, molecular mass markers GeneRulerTM DNA Ladder Mix (Thermo Scientific). (PDF) [file pone.0289976.s005.pdf]

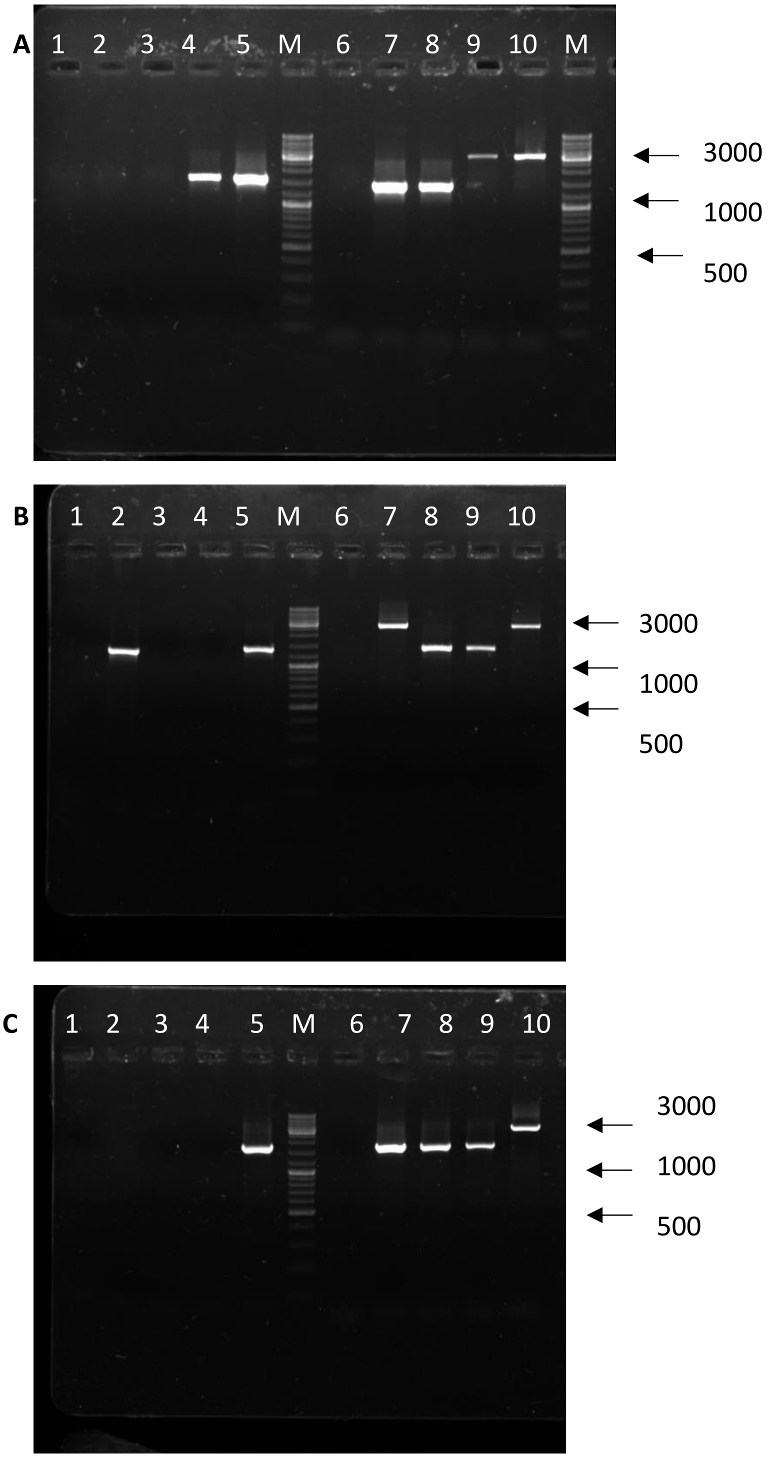

S1 Fig. Gel electrophoresis of PCR products obtained with the primers fumI-F and fumI-R for the *fumI* gene (A, line 1 – 5), fumI-F-up and fumI-down-R for the up-down fragment of the *fumI* gene (A, lines 6 – 10), fumC-F and fumC-R for the *fumC* gene (B, lines 1 – 6), fumC-F-up and fumC-down-R for the up-down fragment of the *fumC* gene (B, lines 6 - 10), Mae-Acc-Nde-F and Mae-Xho-R [1] for the *sfc* gene (C, lines 1 – 5), mae-F-up and mae-down-R for the up-down fragment of the *mae* gene (C, lines 6 - 10) using template DNA: from the *ΔmaeΔfumI* strain (lanes 2, 7), *ΔmaeΔfumIΔfumC* strain (lanes 3, 8), *ΔmaeΔfumC* strain (lanes 4, 9), the wild type strain (lane 5, 10). Lines 1 and 6 were negative control without DNA; M, molecular mass markers GeneRulerTM DNA Ladder Mix (Thermo Scientific).

1. Rozova ON, Mustakhimov II, But SY, Reshetnikov AS, Khmelenina VN. Role of the malic enzyme in metabolism of the halotolerant methanotroph *Methylotuvimicrobium alcaliphilum* 20Z. PLoS One. 2019;14(11):e0225054. doi: 10.1371/journal.pone.0225054.
