# Supplementary material for: Interchangeability of class I and II fumarases in an obligate methanotroph Methylotuvimicrobium alcaliphilum 20Z
Source: PLoS One. 2023 Oct 26;18(10):e0289976. doi: 10.1371/journal.pone.0289976 (PMC10602362; doi:10.1371/journal.pone.0289976)
Supplement: S2 Fig — Velocity versus substrate curves of the M. alcaliphilum FumI (A, C, E) and FumC (B, D) toward malate (A, B), fumarate (C, D), and mesaconate (E) as a substrate. Vmax is the maximum velocity of the reaction, [S] is the substrate concentration, Km is the concentration of the substrate when the reaction velocity is half of Vmax, n is Hill coefficient, S0.5 is the substrate concentration at half Vmax when n ≠ 1. (PDF) [file pone.0289976.s006.pdf]

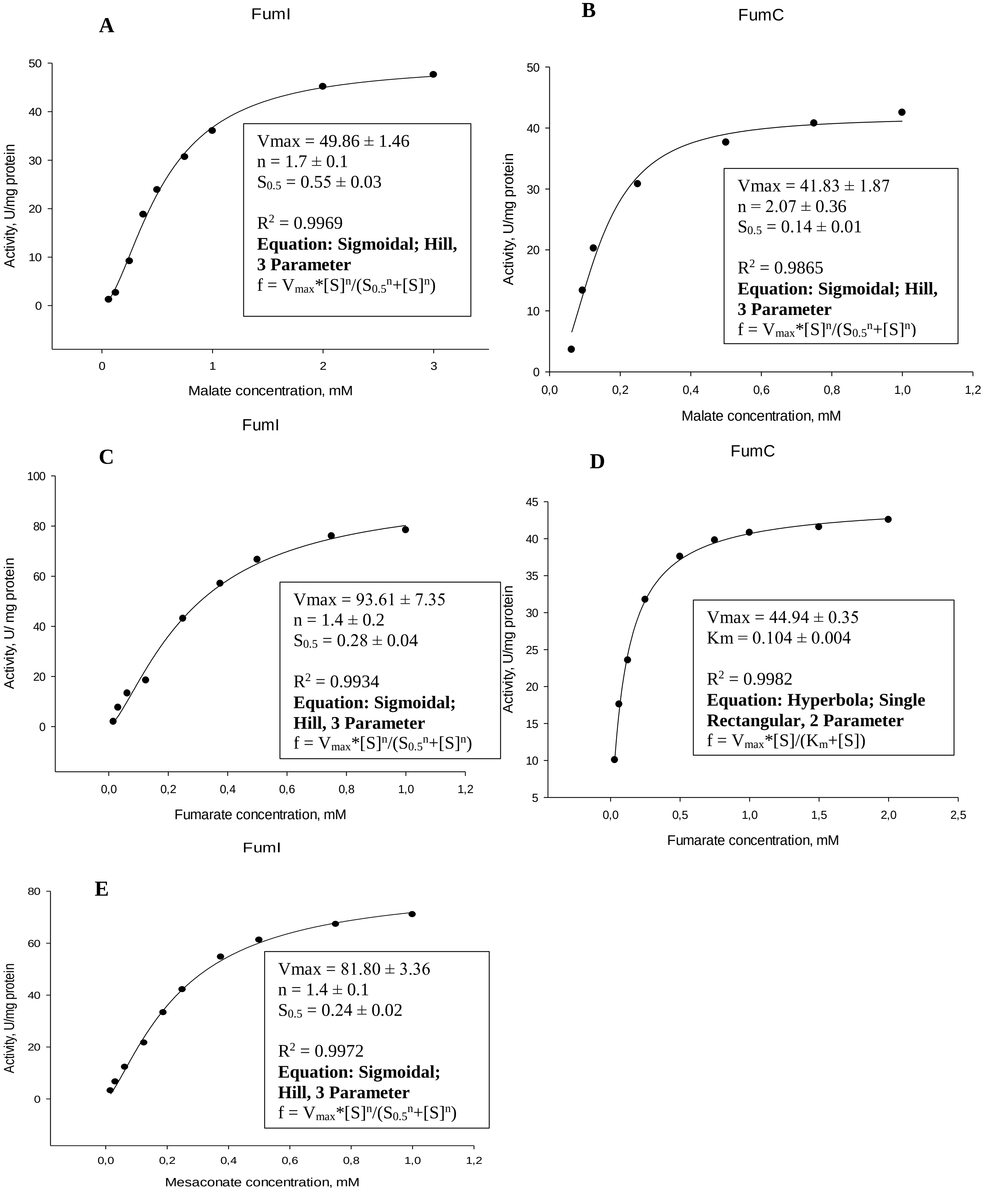

S2 Fig. Velocity versus substrate curves of the *M. alcaliphilum* FumI (A,C,E) and FumC (B,D) toward malate (A,B), fumarate (C,D), and mesaconate (E) as a substrate. V_{max} is the maximum velocity of the reaction, *[S]* is the substrate concentration, K_m is the concentration of the substrate when the reaction velocity is half of V_{max}, n is Hill coefficient, $S_{0.5}$ is the substrate concentration at half V_{max} when $n \neq 1$.
