# Supplementary material for: Interchangeability of class I and II fumarases in an obligate methanotroph Methylotuvimicrobium alcaliphilum 20Z
Source: PLoS One. 2023 Oct 26;18(10):e0289976. doi: 10.1371/journal.pone.0289976 (PMC10602362; doi:10.1371/journal.pone.0289976)
Supplement: S3 Fig — The asterisk indicates amino acid residues forming active site. (PDF) [file pone.0289976.s007.pdf]

```
             1                                                                                               100
Mtm.acl (1)  -------------------------------------------MTLIRQEDFIQSIFESL----QYISYYHPQDFIEAMYRAYQN---------------
B.xenov (1)  ------------------------------VSDNKRPVSTELAMTVIKQEDLIQSIADSL----QYISYYHPLDYIQALGRAYEL---------------
L.major (1)  MSLCDQCEIGCRRVGIKDIEDASAVNADFHFSAIFQPTDPHHHQTEFAKVEGSEKYVEEVEVFGRQALKVNPEALTILAHRAFSDVHHFFRKDHLEGWRR
P.therm (1)  -----------------------------------------------MRTINTSVITENVARLCQEANFNLGHDVMEAFKKAQEE---------------
E.coli  (1)  ------------------------MSNKPFIYQAPFPMGKDNTEYYLLTSDYVSVADFDG----ETILKVEPEALTLLAQQAFHDASFMLRPAHQKQVAA
Consensus                                        P       T I   D I  I E L    Q I Y  P DLI A  RAF D
             101                             ***                                         *                    200
Mtm.alc (39) -----EQSSAAKDAIAQILINSRMCAEGHRPICQDTGIVVVFLKIGMNVQWDAQISI-TDMVNEGVRRAFMHPDNILRASILSDPAGKRINTRDNTPAVV
B.xenov (52) -----EQSPAAKDAIAQILTNSRMCAEGKRPICQDTGIVTVFVKVGMDVRWDGATMGVTDMINEGVRRGYLNPDNVLRASIVSPPEGGRKNTKDNTPAVI
L.major(101) AIEDPEASDNDRYVATTLLKNACIAAGRVLPSCQDTGTAIVLGKRGELCWTGGEDE---KYLSKGIWNAYRY-HNLRYSQTAALDMFKECNTGDNLPAQL
P.therm (39) -----EISLTGKGILQDLIVNAEIACEEQAPMCQDTGYAVIFLELGQDVRIEGGDLY--EAINEGVRKGYTE---GYLRKSIVGHPLERKNTGDNTPAVI
E.coli  (73) ILHDPEASENDKYVALQFLRNSEIAAKGVLPTCQDTGTAIIVGKKGQRVWTGGGDE---ETLSKGVYNTYIE-DNLRYSQNAALDMYKEVNTGTNLPAQI
Consens(101)      E S  AK VI QIL NS IAAEG  PICQDTG AIVFLKIG  V  DGGD    D INEGVRRAYI  DNL  A IA     KR NTGDNTPAVI
             201              *                                     *                                         300
Mtm.alc(133) YMEVVPGDRVEVEIAAKGGGSEAKS----KFAMLNPSDSIVDWVMQTVPTMGAGWCPPGILGIGIGGT-AEKAMLLAKQACMESVDIHELIARGPVNALE
B.xenov(147) HYEIVPGNTVDVQVAAKGGGSENKS----KFAMLNPSDSIVDWILKTVPTMGAGWCPPGMLGIGIGGT-AEKAMVMAKESLMDPIDIQDVIARGPKDWIE
L.major(197) DLLAVPGSDYEFLFIAKGGGSANKAYLYQETKALLNPKSLRAFIEEKLKTLGTAACPPYHIALVIGGTSAEMTMKTVKLASCRYYDSLPTTGDKYGRAFR
P.therm(129) HTKIVPGDKLRIIVAPKGGGSENMSAI-KMLRPADGVEGVKKFVIDTVKAAGPNPCPPIVVGVGIGGT-FEKAAMLAKEALLRELGEKSKYPD--IAKLE
E.coli (169) DLYAVDGDEYKFLCVAKGGGSANKTYLYQETKALLTPGKLKNFLVEKMRTLGTAACPPYHIAFVIGGTSAETNLKTVKLASAHYYDELPTEGNEHGQAFR
Consens(201) HL IVPGD VEVLVAAKGGGSENKS L    K L   DSIK FIIETVKTLG A CPP IIGIGIGGT AEKAMLLAK A M  ID     A     ALE
             301                                   *           *                                              400
Mtm.alc(228) ELRLE--LYEKVNALGIGAQGMGGLTTVLDIKIKDYPAHAANKPVAMIPNCAATRHIHFELDGSGPAELPV---PCLEDWPKIAWEGGNS-AKRVNLDTV
B.xenov(242) ELRVE--LHEKVNALGIGAQGLGGLATVLDVKIMAAPTHAASKPVAIIPNCAATRHAHFTLDGSGAARLEA---PSLDAWPKVHWQPDTEKSKRVDLNTL
L.major(297) DPEWEKIVMEVAQKSGIGAQ-FGGKYFAHQARVIRLPRHGASCPVGLAVSCSADRQILAHINKSGIYIEQLEQNPAQYLPDIPEVHLSTT-SVKVDLKRP
P.therm(225) EELLS----E-INKLGIGPQGLGGKTTALGVHIEIYAAHIASLPVAVNLNCHAARHKEVTMKCLTQIRLTT------------------------PLNDD
E.coli (269) DVQLEQELLEEAQKLGLGAQ-FGGKYFAHDIRVIRLPRHGASCPVGMGVSCSADRNIKAKINREGIWIEKLEHNPGQYIPQELR-QAGEGEAVKVDLNRP
Consens(301) EL LE  L E VNKLGIGAQGLGGK TALDIKII  P HAAS PVAM VNCAA RHI   I  SG   L L   P       I        A KVDLN
             401                       *                                               **  *                  5*0
Mtm.alc(322) TKD---EIATWRPGETLLLSGTLLTGRDAAHKRIVEMLERGEALPEGVDFTNKFIYYVGPVDPVRDEVVGPAGPTTATRMDKFTEAMLAQTGLIGMIGKA
B.xenov(337) TPE---EVAAWTPGQTLLLSGKMLTGRDAAHKRIADMLAKGEKLP--VDFTNRVIYYVGPVDPVRDEAVGPAGPTTATRMDKFTEMMLAQTGLISMIGKA
L.major(395) IDKVRQQLSQYPVGTRVMLNGTLIVARDIAHAKIKEMMDNGEPLPEYMK--TSPIYYAGPAKTPEGYASGSFGPTTAGRMDSYVDLFQSHGGSYITLAKG
P.therm(296) VVE---KL---RIGQRVLISGKIYTGRDAAHKRLVDLIDQGKELP--FDPKGQIIYYVGPTPAKPGKVIGSCGPTTSYRMDPYAPKLYA-LGLKATIGKG
E.coli (367) MKEILAQLSQYPVSTRLSLTGTIIVGRDIAHAKLKELIDAGKELPQYIK--DHPIYYAGPAKTPAGYPSGSLGPTTAGRMDSYVDLLQSHGGSMIMLAKG
Consens(401) I E    LA W VG RLLLSGTIITGRDAAHKRI EMID GE LP  VD     IYYVGP     G  VGS GPTTA RMD Y DLL A  GLI MIGKG
             501                                                                                    590
Mtm.alc(419) ERGPVAIEAIKKHRAVYLMAVGG-AAYLVSKAIRKARVVAFEDLGMEAVHEFVIEDMPVTVAVDVDGESIHKSGPKQWQAKIGKIPVVSK
B.xenov(432) ERGPVAIEAIRKHKAAYLMAVGG-AAYLVSKAIRSAKVLAFEDLGMEAIYEFDVQDMPVTVAVDSNGTSVHQTGPKEWQARIGKIPVATV
L.major(493) NRSKQVTDACKKHGGFYLGSIGGPAAILAKDSIKQVTCLAFPELGMEAVWKIEVEDFPAFIVVDDKGNDMYSKTLA--------------
P.therm(387) RRSPEVIKAMKQYKGVYLAAIGG-AAALIARCIKDAKVIAYPELGPEAIHELVVEDLPVIVVNDTLGGDLYDEGIKIYGVK---------
E.coli (465) NRSQQVTDACHKHGGFYLGSIGGPAAVLAQQSIKHLECVAYPELGMEAIWKIEVEDFPAFILVDDKGNDFFQQIVNKQCANCTK------
Consens(501)  RSP VIDAIKKHKG YLGAIGG AA LVSKAIK AKVLAFPELGMEAIWEIEVEDMPV VVVD  G DIH  GIK W AK  K
```

S3 Fig. Multiple alignment of the primary structure of class I fumarase: Mtm. alc (CCE23513.1), L.major (CBZ12536.1), P.therm (BAF59537.1), B.xenov (ABE29843.1), E.coli (BAE78124.1). The asterisk indicates amino acid residues forming active site.
