# Supplementary material for: Interchangeability of class I and II fumarases in an obligate methanotroph Methylotuvimicrobium alcaliphilum 20Z
Source: PLoS One. 2023 Oct 26;18(10):e0289976. doi: 10.1371/journal.pone.0289976 (PMC10602362; doi:10.1371/journal.pone.0289976)
Supplement: S4 Fig — The growth of M. alcaliphilum 20Z (red line), and mutant strains with genotype ΔmaeΔfumIΔfumC (green line), ΔmaeΔfumI (blue line) and ΔmaeΔfumC (yellow line) on methane (A) and methanol (B) in the presence of 3% NaCl. (PDF) [file pone.0289976.s008.pdf]

A

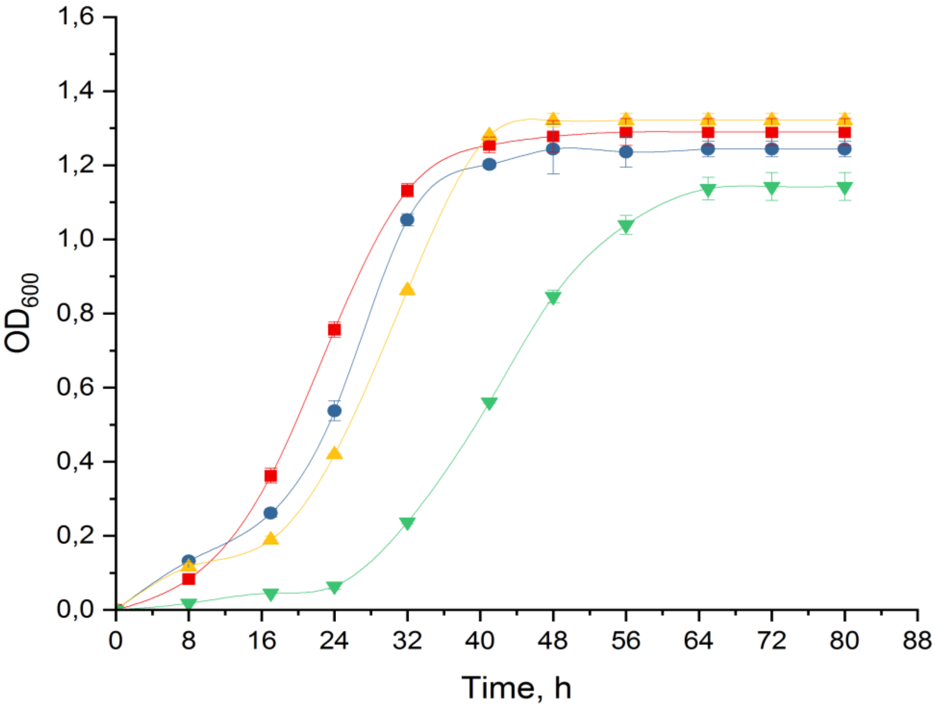


B

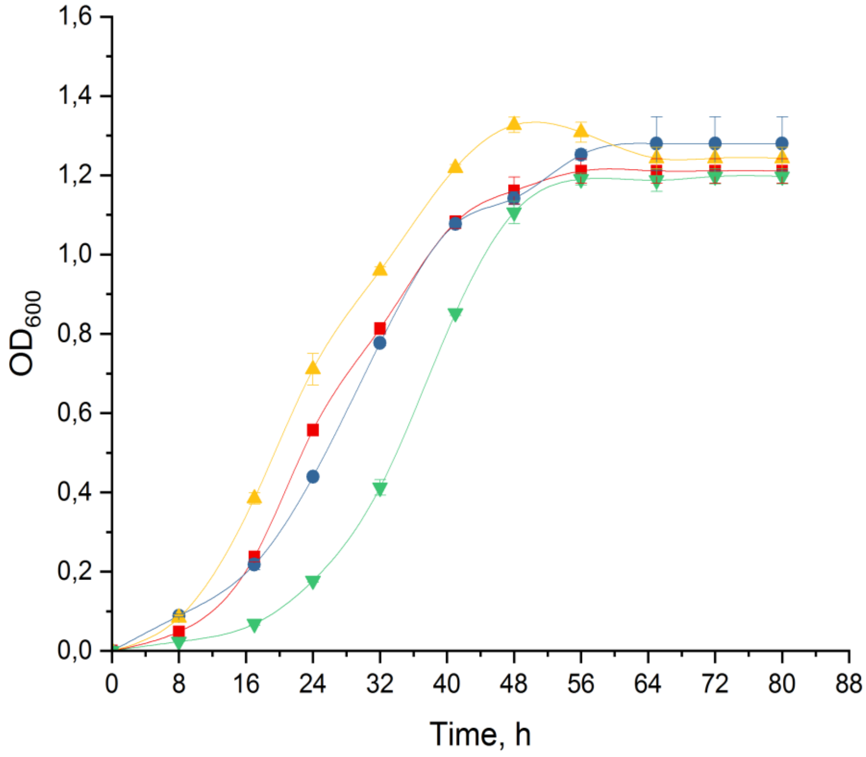


**S4 Fig**. The growth of *M. alcaliphilum* 20Z (red line), and mutant strains with genotype *ΔmaeΔfumIΔfumC* (green line), *ΔmaeΔfumI* (blue line) and *ΔmaeΔfumC* (yellow line) on methane (A) and methanol (B) in the presence of 3% NaCl.
