# Supplementary material for: Interchangeability of class I and II fumarases in an obligate methanotroph Methylotuvimicrobium alcaliphilum 20Z
Source: PLoS One. 2023 Oct 26;18(10):e0289976. doi: 10.1371/journal.pone.0289976 (PMC10602362; doi:10.1371/journal.pone.0289976)
Supplement: S5 Fig — There were a total of 565 positions in the final dataset. Tree topography and evolutionary distances are given by the neighbor-joining method with Poisson correction. The scale bar represents a difference of 0.1 substitutions per site. The characterized enzymes are in bold [1, 8, 10, 12–15]. The amino acid accession numbers in the GeneBank are in brackets. Alpha-proteobacterial methanotrophs are brown, gamma-proteobacterial methanotrophs are violet. (PDF) [file pone.0289976.s009.pdf]

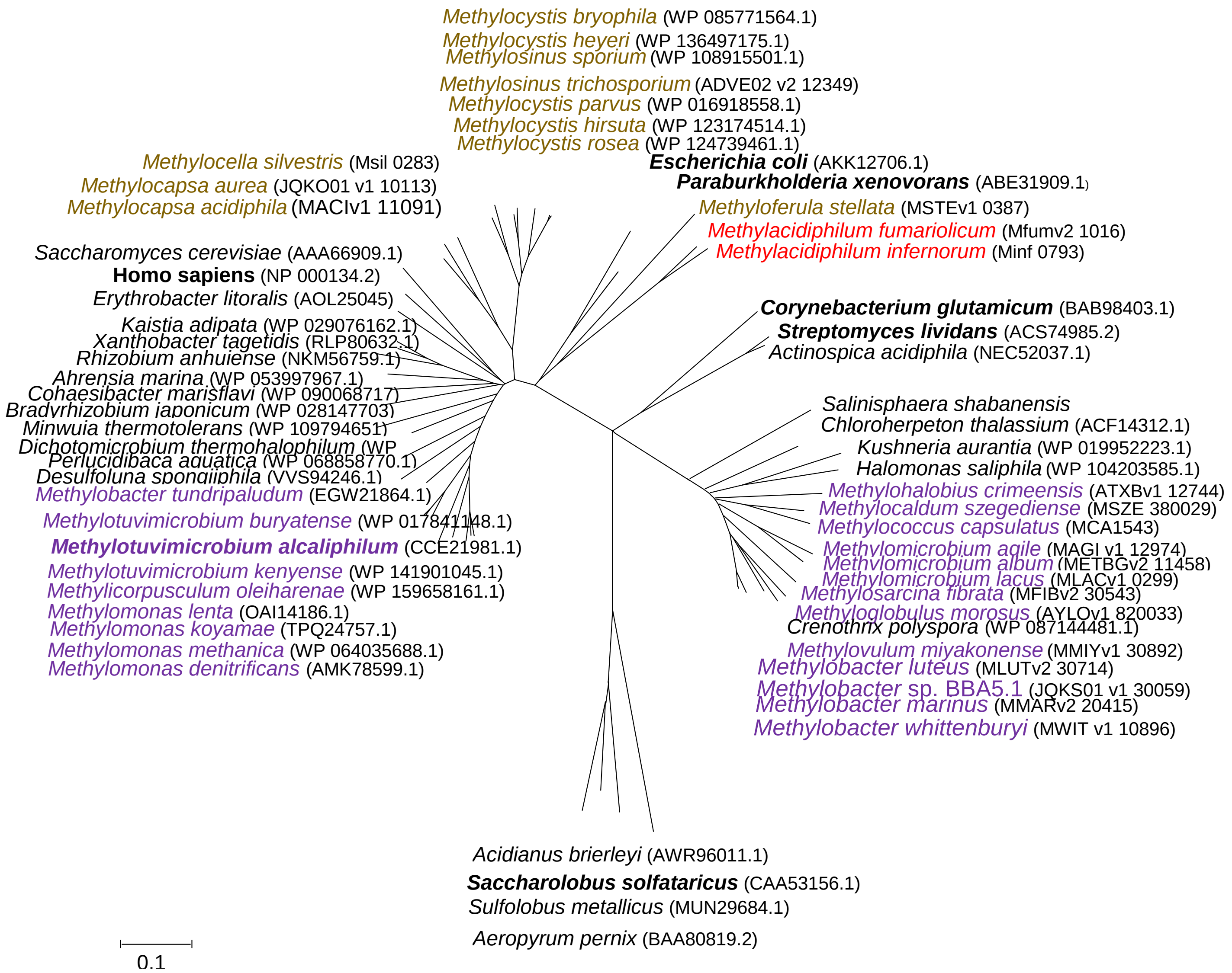

S5 Fig. Phylogenetic tree of class II fumarases. There were a total of 565 positions in the final dataset. Tree topography and evolutionary distances are given by the neighbor-joining method with Poisson correction. The scale bar represents a difference of 0.1 substitutions per site. The characterized enzymes are in bold [1-7]. The amino acid accession numbers in the GeneBank are in brackets. Alpha-proteobacterial methanotrophs are brown, gamma-proteobacterial methanotrophs are violet.

References

1. Kronen M, Sasikaran J, Berg IA. Mesaconase Activity of Class I Fumarase Contributes to Mesaconate Utilization by *Burkholderia xenovorans*. Appl Environ Microbiol. 2015, 81: 5632-8. doi: 10.1128/AEM.00822-15.
2. Estévez M, Skarda J, Spencer J, Banaszak L, Weaver TM. X-ray crystallographic and kinetic correlation of a clinically observed human fumarase mutation. Protein Sci. 2002 Jun;11(6):1552-7. doi: 10.1110/ps.0201502. PMID: 12021453
3. Genda T, Watabe S, Ozaki H (2006) Purification and Characterization of Fumarase from *Corynebacterium glutamicum*, Bioscience, Biotechnology, and Biochemistry, 70:5, 1102-1109, DOI: 10.1271/bbb.70.1102
4. Woods SA, Schwartzbach SD, Guest JR. Two biochemically distinct classes of fumarase in *Escherichia coli*. Biochim Biophys Acta. 1988 Apr 28;954(1):14-26. doi: 10.1016/0167-4838(88)90050-7. PMID: 3282546.
5. Ueda Y, Yumoto N, Tokushige M, Fukui K, Ohya-Nishiguchi H. Purification and characterization of two types of fumarase from *Escherichia coli*. J Biochem. 1991 May;109(5):728-33. doi: 10.1093/oxfordjournals.jbchem.a123448. PMID: 1917897.
6. Su RR, Wang A, Hou ST, Gao P, Zhu GP, Wang W. Identification of a novel fumarase C from *Streptomyces lividans* TK54 as a good candidate for L-malate production. Mol Biol Rep. 2014 Jan;41(1):497-504. doi: 10.1007/s11033-013-2885-8. Epub 2013 Dec 5. PMID: 24307253.
7. Puchegger S, Redl B, Stöffler G. Purification and properties of a thermostable fumarate hydratase from the archaeobacterium *Sulfolobus solfataricus*. J Gen Microbiol. 1990 Aug;136(8):1537-41. doi: 10.1099/00221287-136-8-1537. PMID: 2124611.
