# Supplementary material for: Interchangeability of class I and II fumarases in an obligate methanotroph Methylotuvimicrobium alcaliphilum 20Z
Source: PLoS One. 2023 Oct 26;18(10):e0289976. doi: 10.1371/journal.pone.0289976 (PMC10602362; doi:10.1371/journal.pone.0289976)
Supplement: S6 Fig — citM–citramalate synthase, leuB–isopropylmalate dehydrogenase, leuCD– 3-isopropylmalate dehydratase/isomerase. (PDF) [file pone.0289976.s010.pdf]

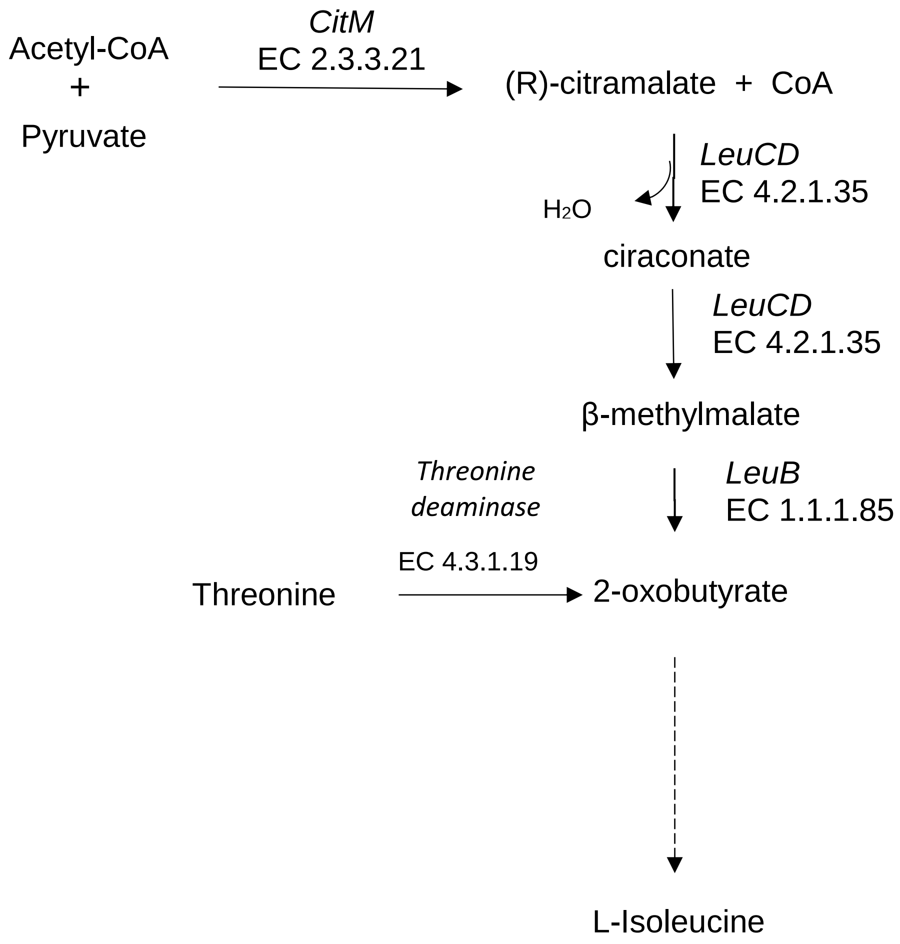

S6 Fig. Pathways for isoleucine biosynthesis in *M. alcaliphilum* 20Z [1,2, KEGG Pathways from www.genoscope.cns.fr]. *citM* – citramalate synthase, *leuB* – isopropylmalate dehydrogenase, *leuCD* – 3-isopropylmalate dehydratase/isomerase.

References

1. Drevland RM, Waheed A, Graham DE. Enzymology and evolution of the pyruvate pathway to 2-oxobutyrate in *Methanocaldococcus jannaschii*. J Bacteriol. 2007 Jun;189(12):4391-400. doi: 10.1128/JB.00166-07. Epub 2007 Apr 20. PMID: 17449626; PMCID: PMC1913355.
2. Risso C, Van Dien SJ, Orloff A, Lovley DR, Coppi MV. Elucidation of an alternate isoleucine biosynthesis pathway in *Geobacter sulfurreducens*. J Bacteriol. 2008 Apr;190(7):2266-74. doi: 10.1128/JB.01841-07.
